# Mechanosensitive super-enhancers regulate genes linked to atherosclerosis in endothelial cells

**DOI:** 10.1083/jcb.202211125

**Published:** 2024-01-17

**Authors:** Jin Li, Jiayu Zhu, Olivia Gray, Débora R. Sobreira, David Wu, Ru-Ting Huang, Bernadette Miao, Noboru J. Sakabe, Matthew D. Krause, Minna U. Kaikkonen, Casey E. Romanoski, Marcelo A. Nobrega, Yun Fang

**Affiliations:** 1 Committee on Molecular Metabolism and Nutrition, Biological Sciences Division, The University of Chicago, Chicago, IL, USA; 2 Department of Medicine, Biological Sciences Division, The University of Chicago, Chicago, IL, USA; 3 Department of Human Genetics, Biological Sciences Division, The University of Chicago, Chicago, IL, USA; 4 A.I.Virtanen Institute for Molecular Sciences, University of Eastern Finland, Kuopio, Finland; 5 Department of Cellular and Molecular Medicine, University of Arizona, Tucson, AZ, USA; 6 Committee on Molecular Medicine, The University of Chicago, Chicago, IL, USA

## Abstract

Vascular homeostasis and pathophysiology are tightly regulated by mechanical forces generated by hemodynamics. Vascular disorders such as atherosclerotic diseases largely occur at curvatures and bifurcations where disturbed blood flow activates endothelial cells while unidirectional flow at the straight part of vessels promotes endothelial health. Integrated analysis of the endothelial transcriptome, the 3D epigenome, and human genetics systematically identified the SNP-enriched cistrome in vascular endothelium subjected to well-defined atherosclerosis-prone disturbed flow or atherosclerosis-protective unidirectional flow. Our results characterized the endothelial typical- and super-enhancers and underscored the critical regulatory role of flow-sensitive endothelial super-enhancers. CRISPR interference and activation validated the function of a previously unrecognized unidirectional flow-induced super-enhancer that upregulates antioxidant genes NQO1, CYB5B, and WWP2, and a disturbed flow-induced super-enhancer in endothelium which drives prothrombotic genes EDN1 and HIVEP in vascular endothelium. Our results employing multiomics identify the cis-regulatory architecture of the flow-sensitive endothelial epigenome related to atherosclerosis and highlight the regulatory role of super-enhancers in mechanotransduction mechanisms.

## Teaser

The role of mechanical forces in blood flow is pivotal in vascular health and the development of diseases like atherosclerosis. This study employs multiomics approaches to elucidate how various flow patterns influence gene regulation in endothelial cells, pinpointing critical super-enhancers involved in mechanotransduction.

## Introduction

Vascular homeostasis and pathology are tightly and dynamically regulated by mechanical forces generated by blood flow (hemodynamics). One unique feature of vascular diseases is that pathological vascular remodelings such as atherosclerosis and stenosis typically occur at sites of curvatures and bifurcations where vascular endothelial cells are activated by disturbed blood flow (DF), which includes features such as flow oscillation, flow reversal, and low time-average shear stress. DF stimulates vascular inflammation, coagulation, and permeability (Davies et al., 2013; Hahn and Schwartz, 2009). In contrast, unidirectional flow (UF) associated with higher time-average shear stress in straight parts of blood vessels promotes endothelial quiescence and barrier integrity (Davies et al., 2013; Hahn and Schwartz, 2009). In addition to vascular diseases, flow-directed mechanotransduction is also instrumental to vasculogenesis and angiogenesis. Blood flow characteristics are major regulators of endothelial transcriptomes (Maurya et al., 2021; Wu et al., 2017). So far, limited functional genetics studies have suggested a critical role of single nucleotide polymorphism (SNP)-imbedded cis-regulatory elements in regulating endothelial transcriptome (Gupta et al., 2017; Krause et al., 2018; Stolze et al., 2020; Wu et al., 2015). Systemic identification of the cis-regulatory elements in endothelial cells under well-defined mechanical forces remains scarce.

Recent epigenetics studies demonstrated that the non-coding human genome is enriched with cis-regulatory elements such as silencers, insulators, and enhancers, each with distinct histone modifications (ENCODE Project Consortium, 2020). Emerging evidence revealed that enhancers orchestrate the cell type–specific patterns of gene expression (Bulger and Groudine, 2010; Heintzman et al., 2009; Heinz et al., 2015) and play key roles in development, evolution, and diseases (Long et al., 2016; Nord et al., 2013; Villar et al., 2015). Enhancers are transcription factor binding site-enriched DNA elements that activate the transcription of a gene from a distance. Direct interaction or looping between enhancers and the promoters of target genes is critical to enhancer function (Deng et al., 2012; Thuijs et al., 2019). The development of DNA sequencing technology prompted genome-wide profiling methods such as ChIP-seq to identify putative enhancers, typically between 10,000 and 150,000 per cell type (Pott and Lieb, 2015). The term “super-enhancer” (SE) emerged to describe putative enhancers in close genomic proximity with a remarkably high degree of enrichment of transcriptional activators or chromatin marks (e.g., H3K27ac) determined by ChIP-seq (Hnisz et al., 2013; Lovén et al., 2013; Whyte et al., 2013). Super-enhancers are typically discovered by a three-step procedure (Pott and Lieb, 2015). In step 1, ChIP-seq peaks are used to define enhancer loci. In step 2, enhancers within 12.5 kb of each other are stitched into a defined single entity spanning a genomic region. In step 3, both stitched and the remaining single enhancers are ranked by the total background-normalized level of the ChIP signal within the genomic region. Super-enhancers (typically <3% of all loci) are defined as enhancer regions that demonstrate ChIP-seq intensity above a cutoff value. For instance, when enhancers are ranked along the x-axis based on the H3K27ac enrichment plotted on the y-axis, super-enhancers are defined as regions to the right of the tangent point (slope = 1) of the resulting curve. The remaining enhancer regions are designated as typical-enhancers (TEs). Previous studies propose that a few hundred of these super-enhancers function as key switches to control cell type–specific gene expression and determine cell fate (Pott and Lieb, 2015). Hnisz et al. employed histone H3K27ac modification to generate a catalog of super-enhancers for 86 human cell and tissue samples and discovered that disease-associated variants are particularly enriched in the super-enhancers of disease-relevant cell types (Hnisz et al., 2013). Super-enhancers are enriched with trait-associated genetic variants and implicated in development (Kai et al., 2021; Lee et al., 2018) and disease progression. However, the typical- and super-enhancer landscape in endothelium under well-defined mechanical cues remains poorly understood.

Integration of epigenomics and human genetics studies has the potential to unveil the molecular underpinning of complex human diseases and discover new gene regulatory mechanisms (Örd et al., 2021). More than 80% of disease-associated alleles found by genome-wide association studies (GWAS) are located in the non-coding genome with undefined functions (Erdmann et al., 2018). Top-scoring disease-associated SNPs are frequently located within cis-regulatory elements, particularly enhancers explicitly active in distinct cell types (Ernst et al., 2011). GWAS have discovered hundreds of common genetic variants significantly associated with cardiovascular diseases (CVDs; Erdmann et al., 2018; Evangelou et al., 2018). The majority of the GWAS-identified CVD SNPs are located in the noncoding genome, with their regulatory mechanism and functional relevance remaining largely unknown. Previously, we reported that rs17114036, a common noncoding polymorphism at 1p32.2 locus strongly associated with coronary artery disease (CAD) and ischemic stroke (IS), is located in a flow-sensitive endothelial enhancer (Krause et al., 2018). Unidirectional flow significantly increases the enhancer activity of rs17114036-containing region to promote the transcription of phospholipid phosphatase 3 (PLPP3, also known as PPAP2B), which maintains the endothelial quiescence and monolayer integrity (Krause et al., 2018; Wu et al., 2015). Systematic identification of CVD SNP-associated endothelial enhancers provides a unique opportunity to unbiasedly identify endothelial cis-regulatory elements prioritized for study and to elucidate novel regulatory mechanisms of CVD-associated variants.

Here, we integrated H3K27ac ChIP-seq, Promoter Capture Hi-C (PCHi-C), transcription factor ChIP-seq, transcriptomics, and human genetics to systematically identify the SNP-enriched cistrome in vascular endothelial cells under well-defined hemodynamics associated with atherosclerosis. A cohort of endothelial typical-enhancers and super-enhancers were identified. We discovered that when compared with typical-enhancers, endothelial super-enhancers are enriched with genetic variants associated with cardiovascular diseases and binding sites for transcription factors key to endothelial functions. These super-enhancers preferentially contact genes controlling endothelial homeostasis and vascular functions. We further identified two distinct cohorts of mechano-sensitive super-enhancers: unidirectional flow (UF)-enriched or disturbed flow (DF)-enriched super-enhancers. Many of these mechanosensitive super-enhancers contain CVD-associated SNP(s), and many of them are physically contacted by promoters of flow-sensitive genes. CRISPR interference and activation validated the enhancer activity of a candidate UF-enriched super-enhancer in promoting the transcription of three antioxidant genes, and a DF-enriched super-enhancer in upregulating two prothrombotic genes in endothelium. To this end, we integrated multilayer omics datasets to systemically characterize the mechanosensitive epigenomic landscape in vascular endothelium under well-defined hemodynamics. These results highlight the regulatory role of CVD SNP-enriched super-enhancers in governing the flow-dependent endothelial transcriptome key to vascular homeostasis and diseases.

## Results

### Endothelial super-enhancers are enriched with transcription factor binding sites and genetic variants associated with cardiovascular diseases

**Figure 1.**
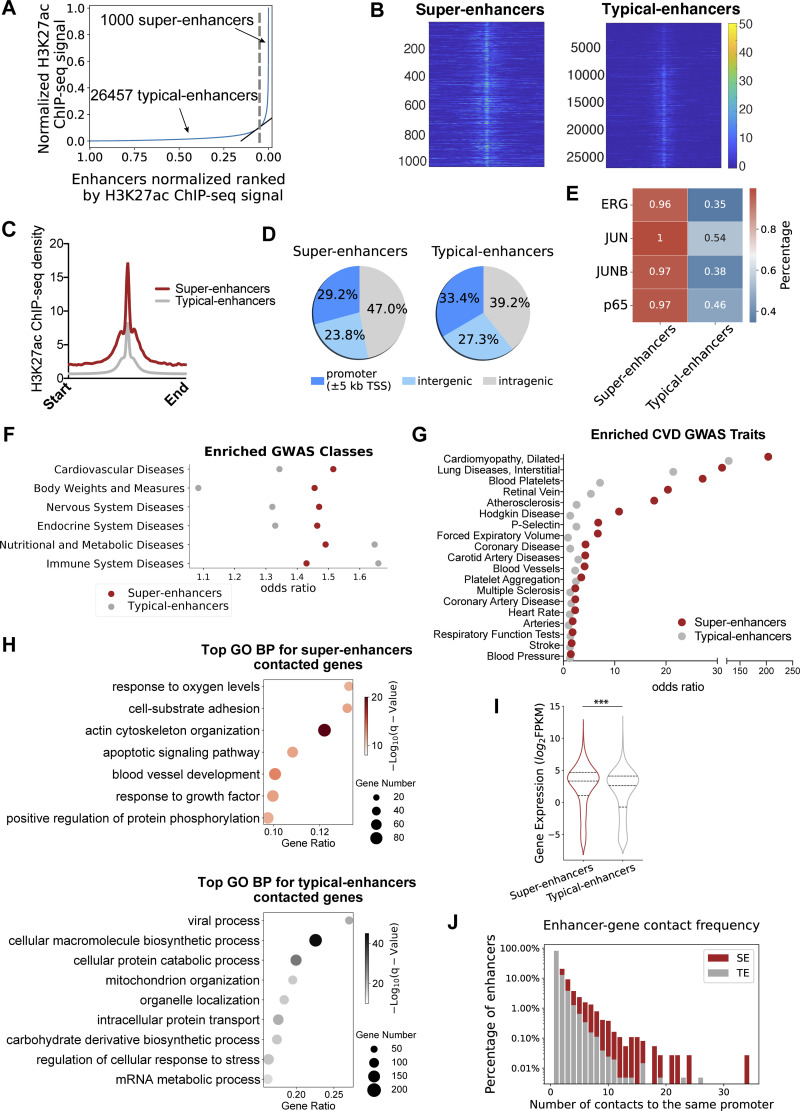
**Endothelial super-enhancers are enriched with transcription factor binding sites and genetic variants associated with cardiovascular diseases and preferentially contact with the promoters of EC-enriched genes. (A)** Typical- and super-enhancers in human aortic endothelial cells (HAECs) are ranked along the x-axis on the basis of H3K27ac enrichment plotted on the y-axis. Super-enhancers (right of the gray dashed line) are defined as regions to the right of the tangency point (slope = 1) of the resulting curve (highlighted in gray). **(B)** Heatmaps of normalized H3K27ac tag counts of the 1,000 super-enhancers (left) and 26,457 typical-enhancers (right) in HAECs. **(C)** Histogram of the averaged normalized H3K27ac tag counts of the 1,000 super-enhancers (red) and 26,457 typical-enhancers (gray) in HAECs. **(D)** Pie charts of genomic distributions of the 1,000 super-enhancers (left) and 26,457 typical-enhancers (right) in HAECs. **(E)** Heatmap demonstrating the percentage of super-enhancers or typical-enhancers containing binding sites for transcription factor ERG, JUN, JUNB, or p65. **(F)** Top GWAS disease classes associated with endothelial SE- or TE-harboring SNPs. X-axis represents the odds ratio of these disease-associated SNPs residing inside versus outside of super-enhancers (red), and inside versus outside of typical-enhancers (gray). **(G)** Top cardiovascular disease (CVD) GWAS traits associated with endothelial SE- or TE-harboring SNPs. X-axis represents the odds ratio of these disease-associated SNPs residing inside versus outside of super-enhancers (red), and inside versus outside of typical-enhancers (gray). **(H)** Gene ontology analyses revealed top biological processes of genes contacted by EC super-enhancers (top) or typical-enhancers (bottom). **(I)** The transcriptional level (FPKM) of genes contacted by super-enhancers (red) or typical-enhancers (gray) in HAECs. Quartiles were represented by dashed lines. **(J)** Gene contact frequency of super-enhancers (red) and typical-enhancers (gray), demonstrated by the percentage of these enhancers that contact with a given promoter. ***P value ≤0.001 was determined by a two-sided Student’s *t* test.

**Figure S1.**
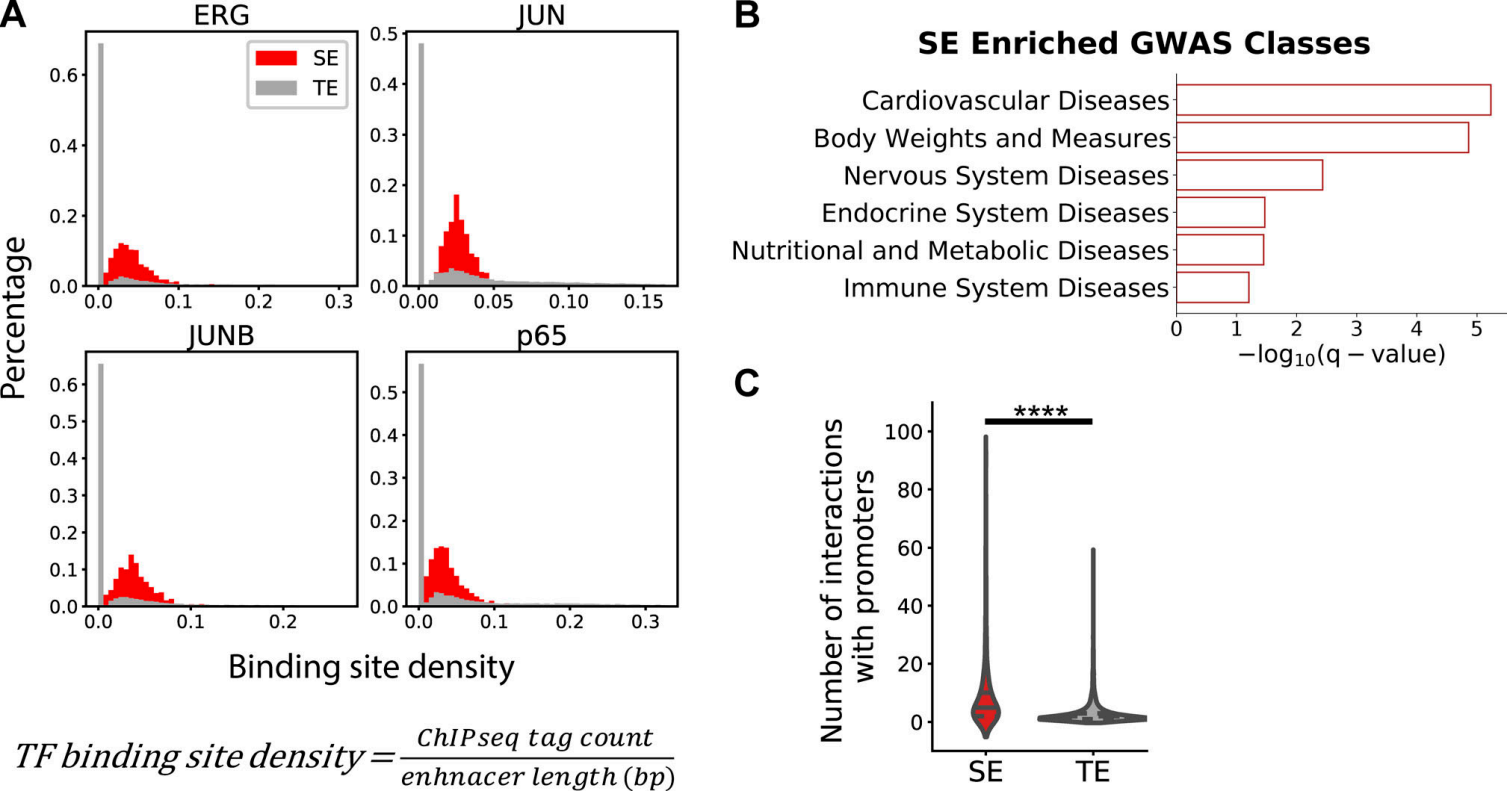
**Endothelial super-enhancers are enriched with EC transcription factor binding sites and cardiovascular disease (CVD) GWAS SNPs and physically contact with more promoters compared with typical-enhancers. (A)** Histograms demonstrating a higher percentage of endothelial super-enhancers (red) than typical-enhancers (gray) containing denser binding sites for transcription factors ERG, JUN, JUNB, and p65. X-axis: transcription factor binding sites density (the ChIP-seq tag counts at each enhancer locus normalized to that enhancer length). Y-axis: percentage of enhancers. **(B)** The top GWAS disease classes associated with endothelial super-enhancers-harboring SNPs, ranked by −log_10_(q value) determined by binomial test. **(C)** PCHi-C in HAECs demonstrate that endothelial super-enhancers form significantly more interactions with promoters than typical-enhancers do. ****P value ≤0.0001 was determined by two-sided Student’s *t* test.

**Figure 2.**
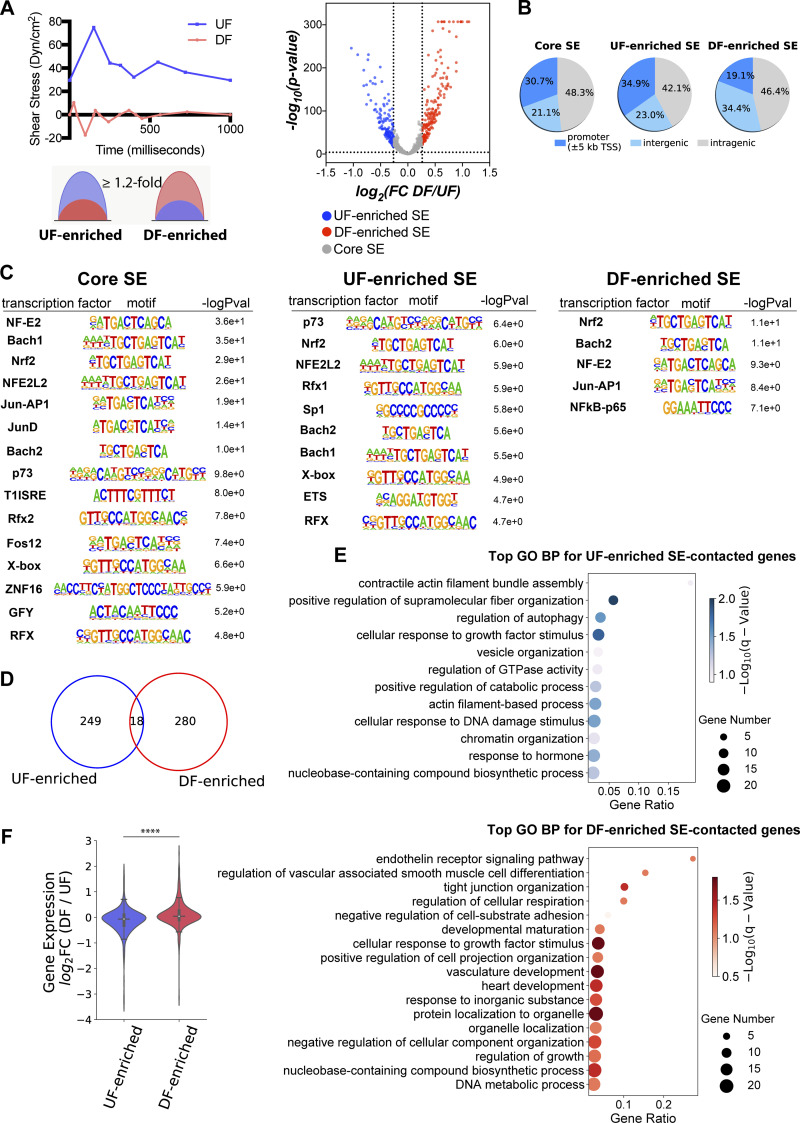
**Flow-sensitive endothelial super-enhancers preferentially contact the promoters of flow-sensitive genes to regulate their transcription. (A)** Classification of UF-enriched super-enhancers and DF-enriched super-enhancers in HAECs. Top left*:* Athero-relevant shear stress was generated by a dynamic flow device and applied to cultured HAECs. Athero-protective UF (blue) and athero-susceptible DF (red) represent hemodynamics in the human distal internal carotid artery and carotid sinus, respectively. Bottom left: Schematic plots depicting UF-enriched or DF-enriched super-enhancers, of which the fold change of H3K27ac signal responding to flows is ≥1.2. Right: volcano plot demonstrating the flow-induced H3K27ac signal change at each super-enhancer locus. UF-enriched super-enhancers (blue dots) are determined by log_2_(fold change, DF/UF) less-than or equal to −0.263 and −log_10_(P value) ≥4; DF-enriched super-enhancers (red dots) are determined by log_2_(fold change, DF/UF) ≥0.263 and −log_10_(P value) ≥4; the complementary sets are defined as core super-enhancers (gray dots) with |log_2_(fold change, DF/UF)| <0.263. **(B)** Pie charts of genomic distributions of core, UF-enriched, and DF-enriched endothelial super-enhancers. **(C)** Enriched transcription factor binding motifs in core, UF-enriched, and DF-enriched endothelial super-enhancers. **(D)** The Venn diagram demonstrating the number of genes uniquely contacted by either UF-enriched or DF-enriched super-enhancers, and genes contacted by both types of super-enhancers. **(E)** Gene ontology analyses revealed biological processes of genes exclusively contacted by either UF-enriched super-enhancers (top) or DF-enriched super-enhancers (bottom). **(F)** Flow-induced endothelial super-enhancers positively correlated with flow-induced transcription. RNA-seq results demonstrate that the majority of genes contacted by DF-enriched super-enhancers have increased transcription levels in HAECs under DF when compared to cells under UF (median log_2_FC > 0, DF/UF), whereas the majority of genes contacted by UF-enriched super-enhancers are transcriptionally upregulated by UF (median log_2_FC < 0, DF/UF). The average transcriptional fold change is significantly different between genes contacted by two clusters of flow-sensitive super-enhancers. Quartiles were represented by black lines. ****P value ≤0.0001 was determined by a two-sided Student’s *t* test.

The whole-genome molecular identity of enhancers in endothelial cells under athero-relevant hemodynamics remains to be determined. We analyzed the data of our H3K27ac ChIP-seq (Krause et al., 2018) conducted in human aortic endothelial cells (HAECs) exposed to either 24-h disturbed flow (DF) or unidirectional flow (UF; Krause et al., 2018). Disturbed flow here recreates the hemodynamics measured in human carotid sinus prone to atherogenesis, and unidirectional flow represents the hemodynamics measured in human distal internal carotid artery resistant to atherosclerosis (Fig. 2 A; Dai et al., 2004). Enhancers within 12.5 kb of each other were stitched together to define a single entity and ranked by increasing the H3K27ac signal to separate SEs from TEs (Hnisz et al., 2013; Whyte et al., 2013). Endothelial enhancers have been systematically identified in cells under static conditions (Brown et al., 2014; Hogan et al., 2017; Stolze et al., 2020), but the epigenome landscape in endothelial cells under flow conditions remains poorly understood. Therefore, we first employed a combined dataset from H3K27ac ChIP-seq conducted in HAEC under UF and in HAEC exposed to DF (Wu et al., 2017). H3K27ac-implicated enhancers were identified separately in HAEC under UF and under DF first and then combined into a list of enhancers in HAEC under flows using HOMER (Heinz et al., 2010). As shown in Fig. 1 A, all enhancers are ranked along the x-axis in the ascending order of H3K27ac signals plotted on the y-axis. Super-enhancers are defined as those to the right of the tangent point (slope = 1) of the resulting curve. In total, we identified 1,000 super-enhancers and 26,457 typical-enhancers in HAECs underflows (Fig. 1 A; and Table S1, a and b). As predicted, the distribution of the overall H3K27ac signal is higher in super-enhancers than in typical-enhancers both at the single locus (Fig. 1 B) and on average (Fig. 1 C). The genome distributions of the typical-enhancers and super-enhancers are described in Fig. 1 D. Recent studies suggested that cell type–specific enhancers are typically activated by unique combinations of a few transcription factors (TFs; Heinz et al., 2015). We next characterized these endothelial SEs and TEs by identifying the binding sites of key transcription factors in the endothelium. Endothelial ChIP-seq results of ERG, JUN, JUB, and NFκB-p65 (Hogan et al., 2017), key transcription factors regulating endothelial homeostasis and inflammation, were employed to identify TF binding sites in these endothelial enhancers. We detected a higher percentage of SEs than TEs containing at least one binding site for these endothelial-enriched TFs (Fig. 1 E). For instance, the binding sites of ERG, a TF regulating endothelial lineage and homeostasis (Shah et al., 2016), were detected in 96% of SEs but only in 35% of TEs. To mitigate the length-dependent bias, as SEs are on average longer in size than TEs, we applied a normalization to calculate the TF binding density by dividing the number of TF ChIP-seq tag counts mapped to each enhancer by its length (bp). The histograms showed that the distribution of SEs was more skewed to the right than TEs for all TFs, suggesting a higher percentage of super-enhancers than typical-enhancers containing denser TF binding sites (Fig. S1 A). These data suggest that in HAECs cultured underflows, endothelial super-enhancers are more enriched with binding sites of key endothelial transcription factors compared with typical-enhancers.

We further examined the enrichment of disease-associated variants in these SEs and TEs using SNPs reported in the NCBI dbGaP (Mailman et al., 2007) and NHGRI GWAS catalogs (Welter et al., 2014). The enrichment analyses were conducted using binomial tests, with the null hypothesis being the probability of observing a single base to be a disease-associated SNP is the same inside as outside of the enhancer regions. These tests demonstrated an enrichment of SNPs associated with a cohort of GWAS categories in both endothelial SEs and TEs (Fig. 1 F). Notably, the odds ratio of CVD SNP presence in SEs (OR = 1.5) is higher than in TEs (OR = 1.3; Fig. 1 F). Further analyses focusing on the cardiovascular diseases (CVD) traits indicated that CVD SNPs are enriched in both types of enhancers and the odds ratios are mostly higher for super-enhancers (Fig. 1 G). Moreover, we detected that CVD, among the GWAS catalog human traits, is the most significantly enriched GWAS class that is associated with SE-harboring SNPs (−*log*_*10*_*[*q value*] =* 5.23, Fig. S1 B). These results support the notion that disease-associated loci are enriched in cis-regulatory regions in corresponding disease-relevant cell types (Hnisz et al., 2013). The enrichment of CVD SNPs (Fig. 1, F and G) along with binding sites of endothelial transcription factors (Fig. 1 E and Fig. S1 A) in endothelial super-enhancers support their putative functions in regulating vascular homeostasis and diseases.

### PCHi-C demonstrated that endothelial super-enhancers preferentially contact with the promoters of EC-enriched genes

Enhancers are proposed to control gene expression by forming physical contacts with target gene promoters, sometimes through long-range chromosomal interactions spanning significant genomic distances (Schoenfelder and Fraser, 2019). Although chromosome conformation capture techniques such as Hi-C (Lieberman-Aiden et al., 2009) have been conducted in adult endothelial cells to probe the genome-wide mapping of long-range chromatin contacts (Åkerborg et al., 2019; Lalonde et al., 2019; Niskanen et al., 2018), most Hi-C datasets have limited resolution (>40 kb) and do not precisely reflect the enhancer–promoter interactions. High-resolution promoter capture Hi-C (PCHi-C) is developed based on Hi-C, but the highly complex libraries were specifically enriched for promoter sequences to identify and interrogate physical interactions between cis*-*regulatory elements and all annotated promoters (Mifsud et al., 2015; Schoenfelder et al., 2015). We conducted PCHi-C in HAECs to generate a three-dimensional (3D) endothelial promoter interactome (Gray et al., 2022). We performed in-situ Hi-C (Montefiori et al., 2018; Rao et al., 2014) to allow enhancer-level resolution of promoter contacts. Computational pipeline CHiCAGO (Cairns et al., 2016), which eliminates sequence capture bias, was employed to identify genomic interactions. PCHi-C captured 114,713 high-confidence interactions (CHiCAGO score ≥5) in HAECs of gene promoters with DNA fragments (Table S1 c). CHiCAGO-analyzed PCHi-C results enabled us to systematically and unbiasedly interrogate the enhancers for their physically contacted promoters/genes.

By integrating the endothelial 3D promoter interactome and the H3K27ac-mapped endothelial enhancers, we discovered that 2,353 promoters are physically contacted by at least one H3K27ac-identified super-enhancers. Meanwhile, 5,669 gene promoters are contacted by at least one TE but not a SE. Gene ontology analyses showed that SE-contacted genes are enriched in endothelium- and vasculature-related biological processes such as response to oxygen, cell-substrate adhesion, and blood vessel development, whereas TE-contacted genes are involved in general cellular pathways such as cellular macromolecule biosynthesis and cellular protein catabolic process (Fig. 1 H). These results agree with the prevailing view that super-enhancers are instrumental in tissue-specific cellular functions (Hnisz et al., 2013; Whyte et al., 2013). Interrogation with the HAEC transcriptome (Wu et al., 2017) demonstrated that genes contacted by SEs are on an average more highly expressed than genes exclusively contacted by TEs (Fig. 1 I). The data support an emerging view that SEs, when compared with TEs, confer higher transcriptional activation on targeted genes (Kalna et al., 2019; Lovén et al., 2013). We also detected that on average an endothelial SE forms significantly more interactions with promoters than a TE does (Fig. S1 C), a phenomenon that was reported in cancer cells (Huang et al., 2018). Moreover, if we only count the contacts to the same promoter, the data showed a rightward skewing of SEs compared with TEs in the histogram (Fig. 1 J), suggesting that a higher proportion of super-enhancers contact with the same promoter repetitively. In other words, different regions within a super-enhancer tend to form simultaneous contacts with their targeted gene. This finding provides a potential mechanism explaining the higher transcriptional regulation activity of SEs than TEs. Together, H3K27ac ChIP-seq, CHiCAGO-analyzed PCHi-C, and transcriptomics support the notion that endothelial super-enhancers are hyperactive regulatory domains that contact with promoters of highly expressed endothelial genes instrumental to key vascular functions. We then prioritized super-enhancers for further analyses and functional validations.

### Unidirectional flow and disturbed flow induce distinct cohorts of endothelial super-enhancers

Mechanical forces are major determinants of the endothelial transcriptome, but the identity of endothelial super-enhancers responding to distinct mechanical cues, such as atherosclerosis-relevant hemodynamics, remains to be determined. We thus set out to systematically identify flow-sensitive endothelial super-enhancers by comparing the H3K27ac ChIP-seq results in HAECs subjected to 24-h athero-protective UF to those in cells exposed to athero-prone DF. We defined a “UF-enriched” super-enhancer when the H3K27ac signal at that super-enhancer locus is greater in HAECs under UF than DF (P value ≤0.0001, Fold Change/FC ≥1.2; Fig. 2 A). Fold change ≥1.2 was chosen based on previous studies, which detected anti-IgM-induced changes of super-enhancers in B cells (FC ≥1.14; Michida et al., 2020) and ERG-dependent endothelial super-enhancers (FC ≥1.5; Kalna et al., 2019). We defined a “DF-enriched” super-enhancer if the H3K27ac signal is greater under DF than UF (P value ≤0.0001, FC ≥1.2; Fig. 2 A). These two clusters were collectively named “flow-sensitive” super-enhancers. In contrast, we defined a “core” super-enhancer when at this given locus, the H3K27ac signal remains similar in HAECs under UF to those under DF. Using these criteria, we classified 152 UF-enriched, 183 DF-enriched, and 665 core super-enhancers in HAECs (Fig. 2 A and Table S2, a–c). The genome distributions of the core and flow-sensitive endothelial super-enhancers are described in Fig. 2 B. We then performed motif analyses to identify the enriched TF binding motifs in each of these three super-enhancer clusters. Common binding motifs of NRF2 (a transcriptional activator) and BACH (a transcriptional suppressor) were detected in both flow-sensitive and core super-enhancers (Fig. 2 C). This implied that both transcriptional activators and transcriptional suppressors may be involved in endothelial super-enhancers activity, which is consistent with the previously reported roles of NRF2 and BACH in driving key endothelial functions such as proliferation, migration, apoptosis, and inflammation (Dai et al., 2007; Jiang et al., 2015). ETS binding motifs are enriched only in the UF-enriched super-enhancers, whereas the binding motifs of NFκB-p65 are only enriched in the DF-enriched super-enhancers (Fig. 2 C). Notably, ETS-1 is induced in endothelial cells under UF (Milkiewicz et al., 2008), and a cooperative action of ETS and KLF2 (a key TF to maintain endothelial quiescence under UF) has been reported (Meadows et al., 2009). Meanwhile, the major role of NFκB-p65 in endothelial inflammation induced by disturbed flow has been well established (Fang et al., 2010; Hajra et al., 2000). These results characterized the flow-sensitive endothelial super-enhancers as a function of hemodynamics critical to the protection or susceptibility to atherosclerosis. These findings also support the proposed model that the combinatorial effects of transcription factor bindings on super-enhancers are key to mediating cell type–specific and context-dependent gene expression in driving cell plasticity (Brown et al., 2014; Hogan et al., 2017).

### Flow-sensitive endothelial super-enhancers physically contact a cohort of promoters, the expression of which is dynamically regulated by hemodynamic forces

**Figure S2.**
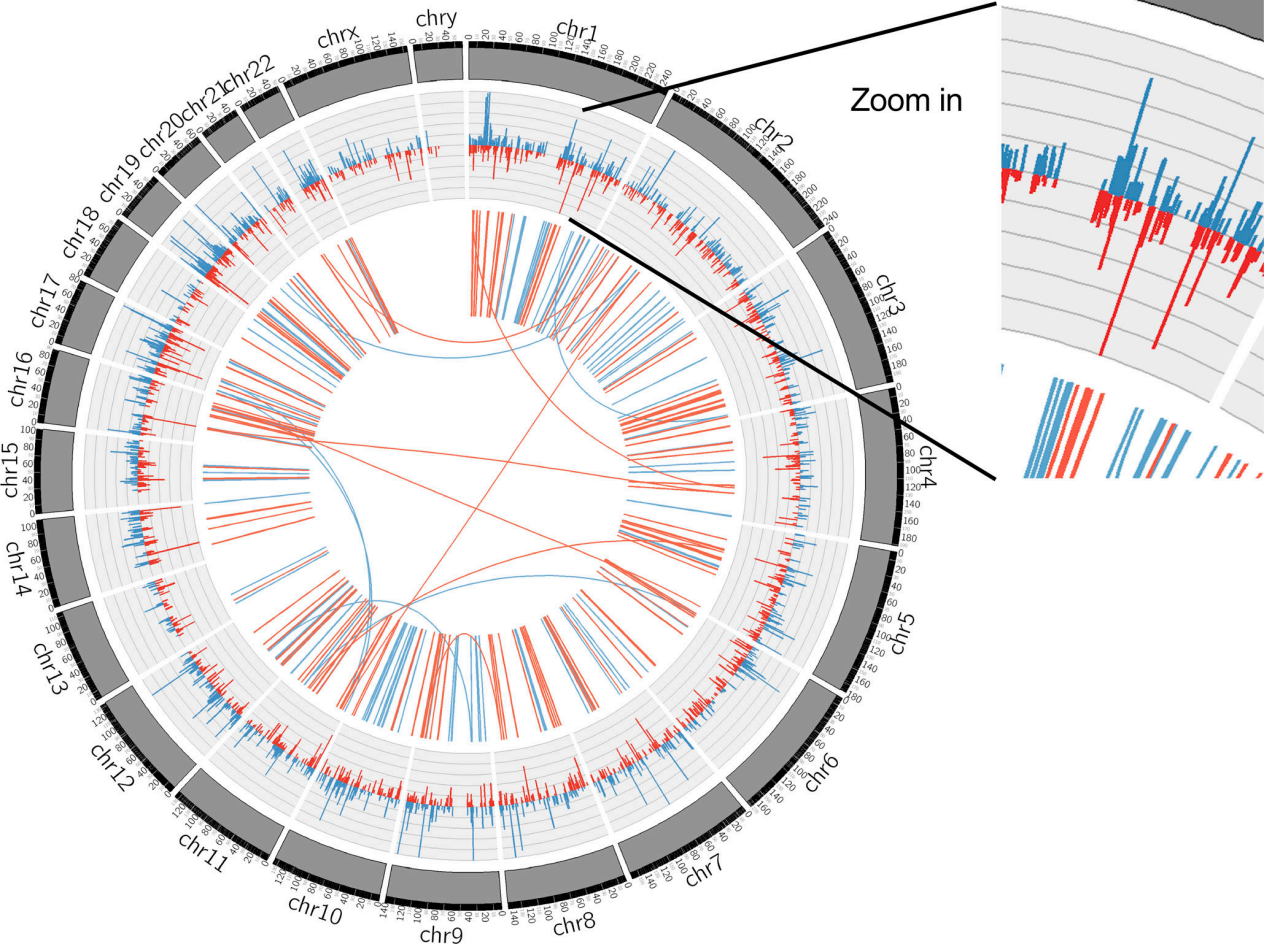
**Circos plot illustrating the genome-wide loci contacted by flow-sensitive super-enhancers and the flow-sensitive transcriptome in HAECs.** Inner circle links: PCHi-C-detected intra- and inter-chromosomal interactions. Physical connections between UF-enriched super-enhancers and gene promoters are labeled in blue, and physical connections between DF-enriched super-enhancers and promoters are labeled in red. Outer circle histograms: RNA-seq-detected expression levels (FPKMs) of flow-sensitive genes (q value ≤0.05) in HAECs subjected to either 24-h UF or DF. Blue histograms represent the mRNA levels of flow-sensitive genes in HAECs cultured under UF and red histograms show their mRNA levels in HAECs cultured under DF. Overall, flow-regulated genes are largely located in genomic loci contacted by flow-sensitive super-enhancers.

To probe possible biological functions of these flow-sensitive endothelial super-enhancers, we employed the PCHi-C results to identify a list of genes whose promoters are physically contacted by UF-enriched or DF-enriched super-enhancers. Among this list, we further selected the genes that are actively expressed in HAECs underflow by integrating with our RNA-seq dataset (Wu et al., 2017) and chose the ones with FPKM ≥1 under either type of flow. In brief, 249 promoters/genes are physically contacted by UF-enriched SEs, whereas 280 promoters/genes are contacted by DF-enriched SEs (Fig. 2 D). Very few (18) promoters are contacted by both UF- and DF-enriched super-enhancers (Fig. 2 D). Gene ontology analyses demonstrated that genes contacted to flow-sensitive SEs participate in biological functions that are fundamental to endothelial mechano-transduction pathways. Genes exclusively contacted by UF-enriched SEs are enriched in biological processes such as actin filament assembly and small GTPase signaling (Fig. 2 E). Genes exclusively contacted by DF-enriched SEs are implicated in the biological functions of the endothelin receptor signaling pathway, tight junction organization, and vasculature development (Fig. 2 E). We next hypothesized that flow-sensitive super-enhancers could coordinately drive flow-sensitive transcriptome. We interrogated the RNA-seq performed in HAECs subjected to either UF or DF and looked up the transcription level of genes contacted by flow-sensitive SEs. RNA-seq results demonstrated that most genes contacted by UF-enriched SEs were also transcriptionally upregulated by UF (median log_2_FC <0, DF/UF), whereas the majority of genes contacted by DF-enriched SEs were upregulated by DF (median log_2_FC >0, DF/UF; Fig. 2 F). The genome-wide loci contacted by flow-sensitive SEs and the flow-sensitive transcriptome in HAECs were plotted in the circos plot (Fig. S2). Inner circle links are PCHi-C-detected intra- and inter-chromosomal interactions. Physical connections between UF-enriched SEs and gene promoters are labeled in blue, and physical connections between DF-enriched SEs and promoters are labeled in red. Most chromosomal interactions between flow-sensitive SEs and endothelial promoters are intrachromosomal, and only a few interactions were interchromosomal. The outer circle histograms represent the mRNA levels (FPKM) of flow-sensitive genes detected by RNA-seq (q value ≤0.05). Blue histograms represent the mRNA levels of flow-sensitive genes in HAECs under UF, and red histograms show their mRNA levels when these cells were under DF. Overall, flow-regulated endothelial genes are largely located in genomic loci contacted by flow-sensitive super-enhancers.

### A cohort of endothelial flow-sensitive super-enhancers that harbor CVD GWAS SNP(s) and contact with gene promoter(s)

**Figure 3.**
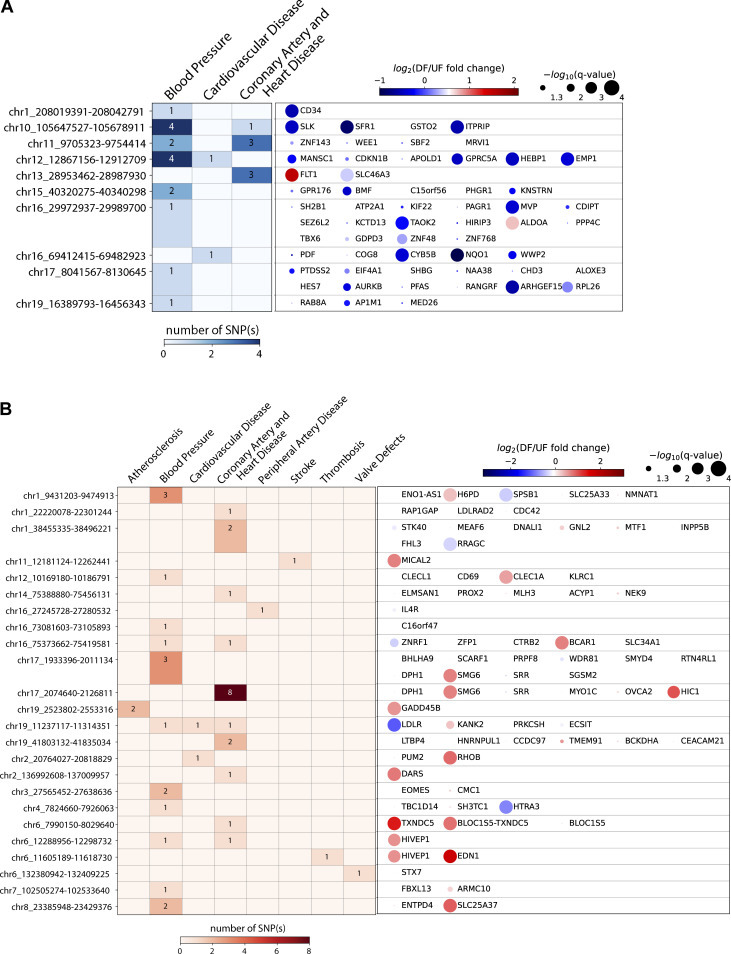
**A cohort of endothelial flow-sensitive super-enhancers harbor CVD GWAS SNP(s) and contact with at least one gene promoter. (A)** Refined UF-enriched endothelial super-enhancers which contain CVD SNP(s) and contact with gene promoter(s). **(B)** Refined DF-enriched endothelial super-enhancers which contain CVD SNP(s) and contact with gene promoter(s). Left heatmaps: The color and number both represent the number of CVD SNP(s) that reside in each super-enhancer locus. The CVD traits associated with these GWAS SNPs are labeled on the top. Right heatmaps: The color of the dots represents the mRNA fold change (log_2_ DF/UF) of each given gene in HAECs as a function of the flows; blue (log_2_FC < 0) highlights genes upregulated by UF and red (log_2_FC > 0) represents genes upregulated by DF. The size of the dots represents the statistical significance (q value) determined in the RNA-seq analysis.

**Figure 4.**
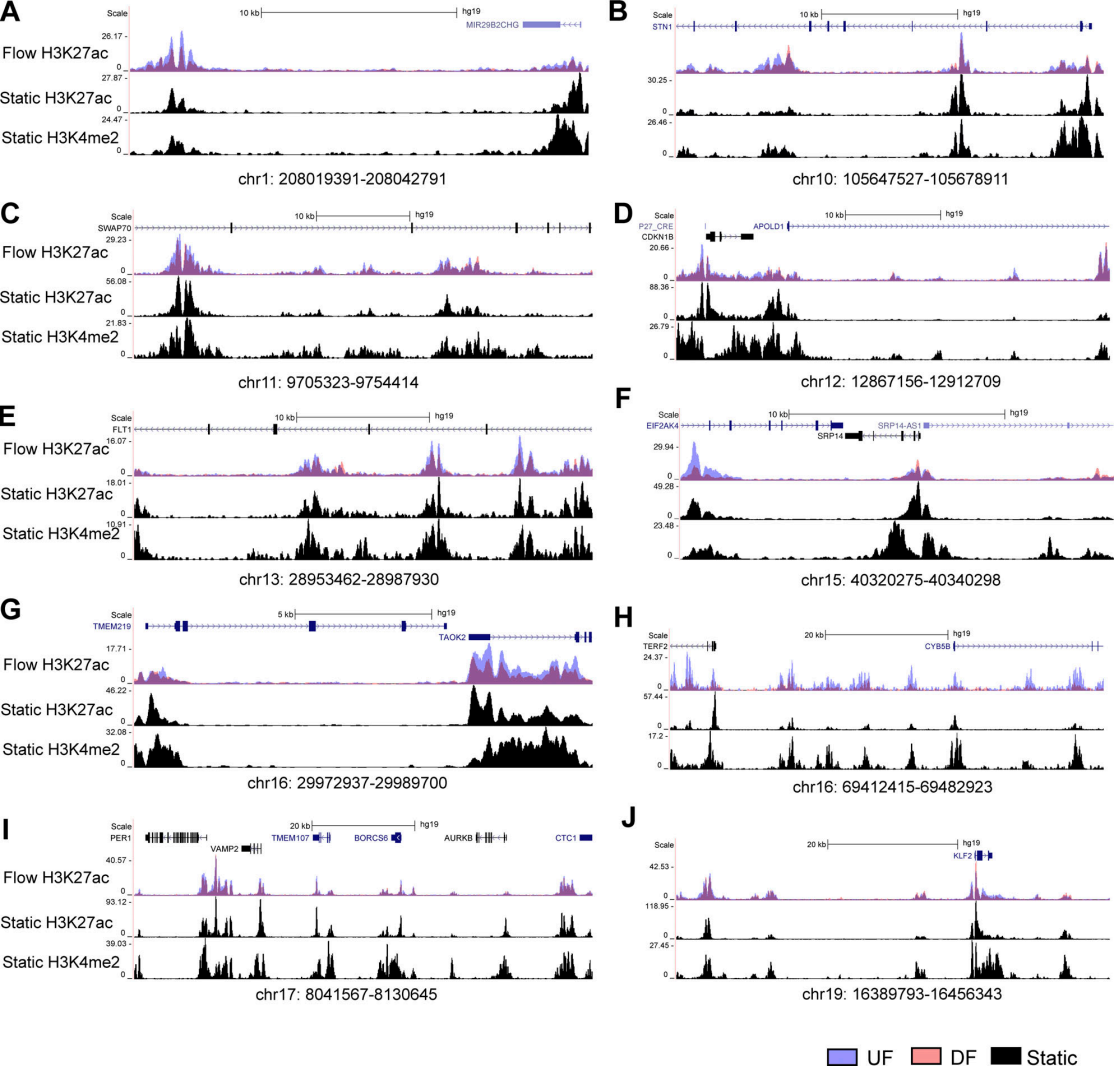
**Genome** **tracks illustrating the 10 UF-enriched super-enhancers are bona fide enhancers in HAECs cultured under static conditions.**

**Figure 5.**
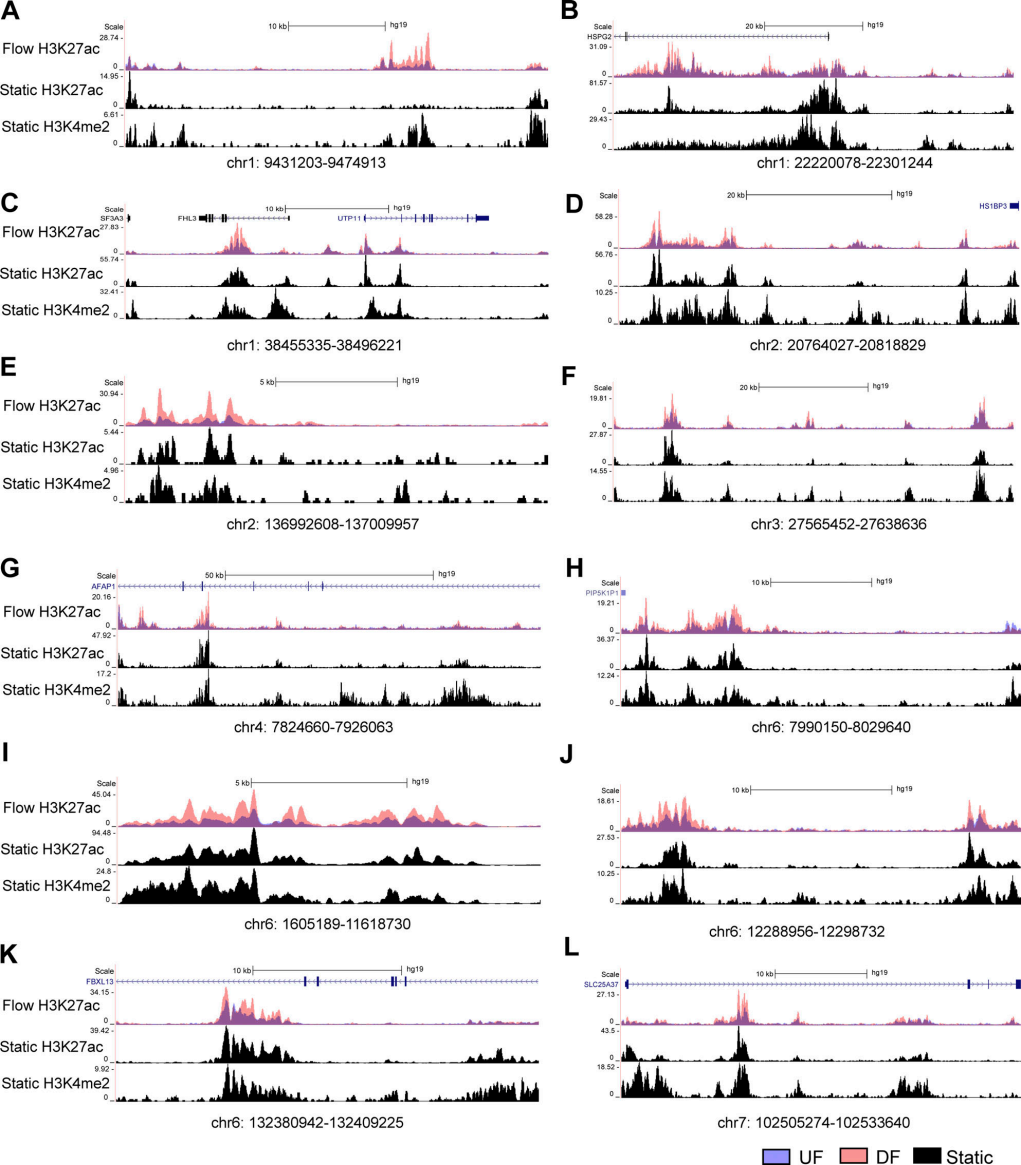
Genome tracks illustrating the first half of the 24 DF-enriched super-enhancers are bona fide enhancers in HAECs cultured under static conditions.

**Figure 6.**
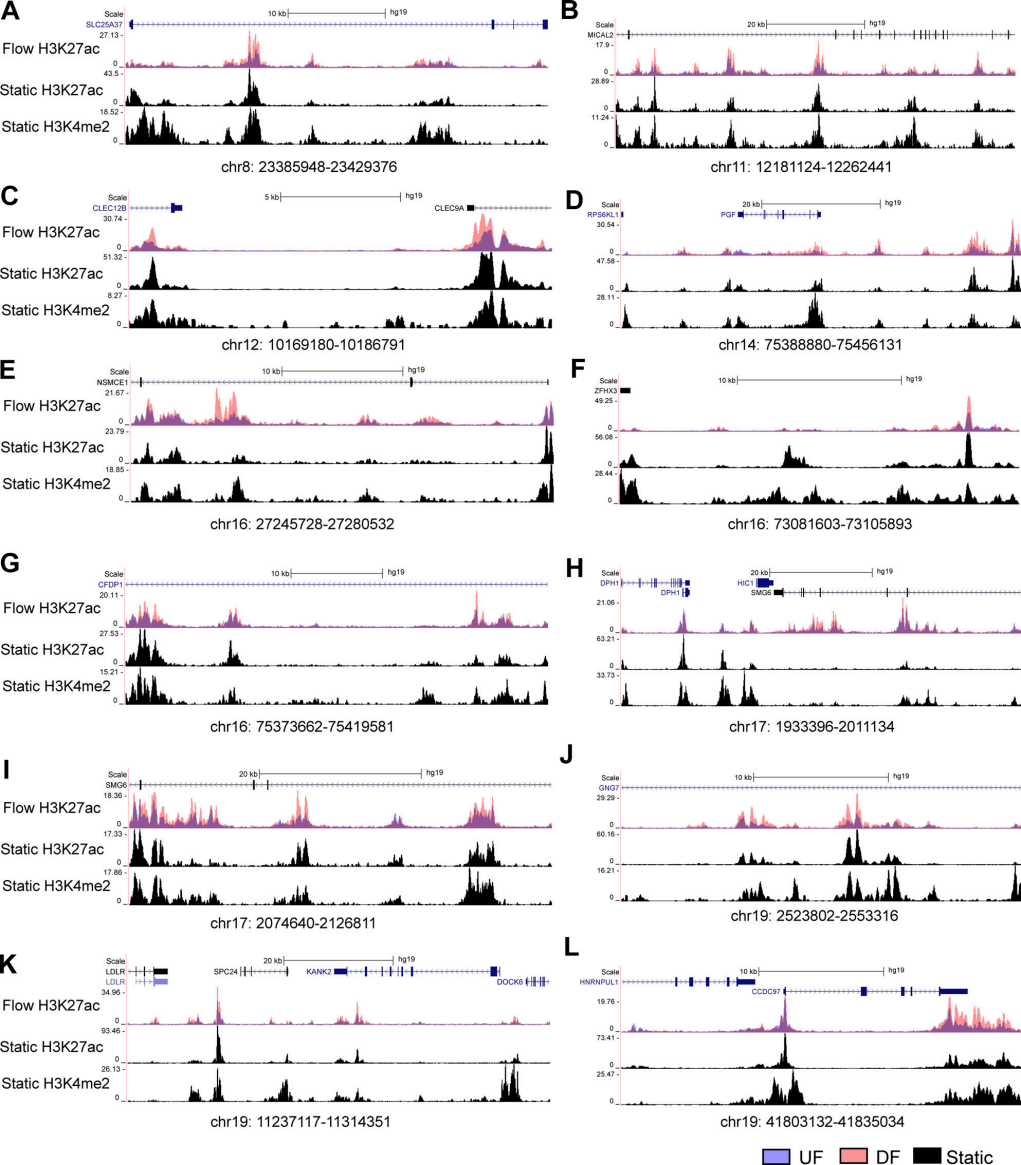
Genome tracks illustrating the second half of the 24 DF-enriched super-enhancers are bona fide enhancers in HAECs cultured under static conditions.

We next refined flow-sensitive endothelial super-enhancers that are likely to have biological functions. We first assessed their enrichment of GWAS SNPs associated with cardiovascular traits curated from NHGRI-EBI GWAS Catalog (Buniello et al., 2019). Subsequently, PCHi-C was used to annotate these CVD SNP-imbedded SEs to their contacted promoters. Our analyses identified 10 UF-enriched and 24 DF-enriched SEs, all of which not only contain at least one cardiovascular trait-associated GWAS SNP but also physically contact at least one gene promoter. Fig. 3, A and B and Table S2, d and e detail the genomic locations of these flow-sensitive endothelial SEs along with the number of imbedded SNPs, their associated CVD traits, and their connected genes. Employing datasets of H3K27ac and H3K4me2 ChIP-seq conducted in HAECs under static conditions (Hogan et al., 2017), we discovered that these 34 super-enhancers are located within enhancer-like elements (Figs. 4, 5, and 6). This suggests that they are bona fide endothelial enhancers, active under both static and flow conditions. Hemodynamic forces appear to be a key determinant of the activity of these mechanosensitive SEs. Interestingly, a majority of these candidate SEs can contact with multiple genes. To further probe the regulatory functions of these CVD SNP-embedded flow-sensitive SEs, we integrated the RNA-seq results and discovered that many of their contacted genes are also transcriptionally regulated by the flow. Particularly, genes contacted by UF-enriched SEs are largely upregulated by UF (Fig. 3 A), whereas those contacted by DF-enriched SEs are mostly transcriptionally elevated by DF (Fig. 3 B). The integration of H3K27ac ChIP-seq, GWAS, PCHi-C, and RNA-seq identified a cohort of CVD SNP-containing flow-sensitive endothelial super-enhancers that physically contact with multiple promoters of flow-sensitive genes.

### UF-enriched super-enhancer chr16: 69412415–69482923 upregulates three UF-induced antioxidant genes NQO1, CYB5B, and WWP2 in HAECs

**Figure 7.**
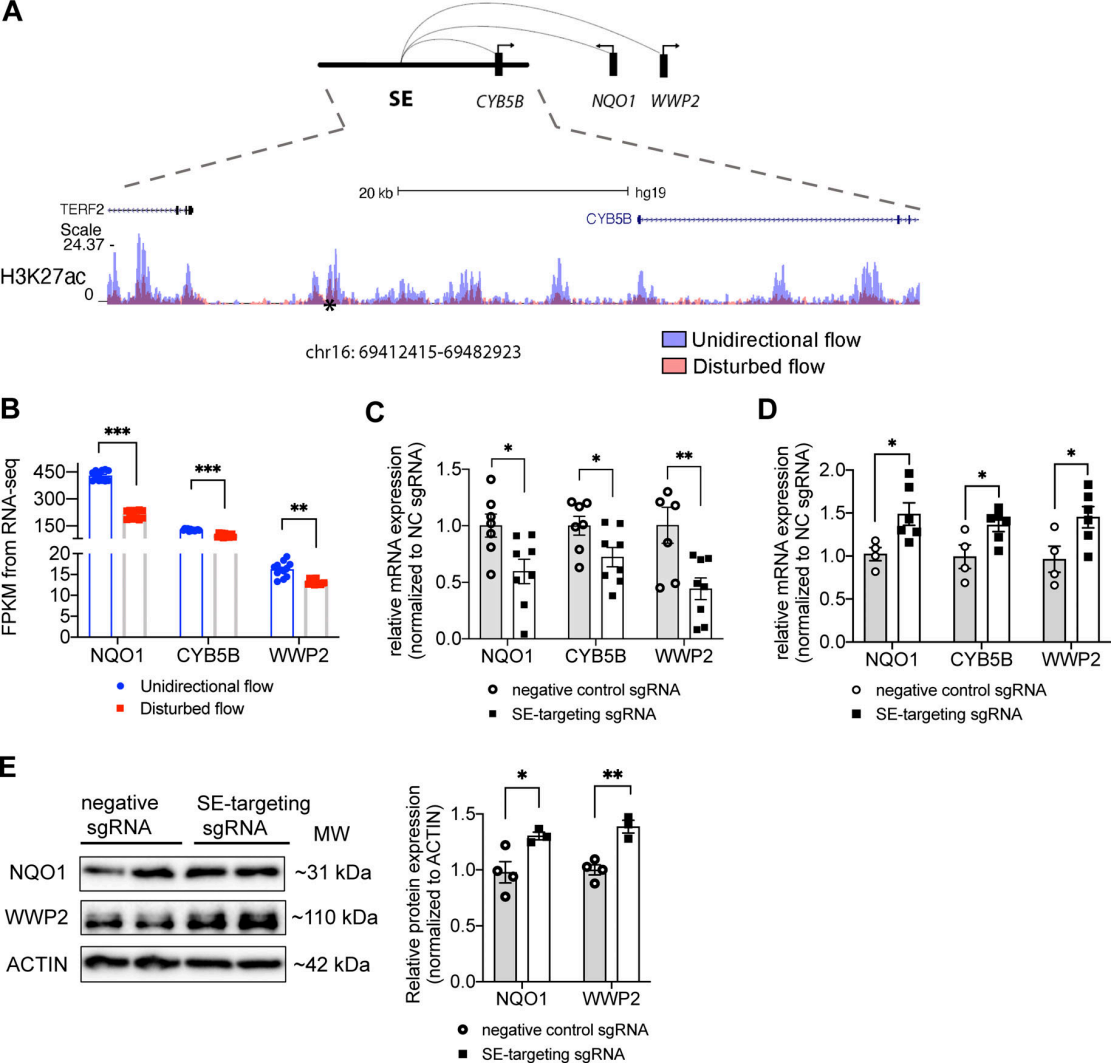
**UF-enriched super-enhancer chr16: 69412415–69482923 up-regulates three UF-induced anti-oxidant genes NQO1, CYB5B, and WWP2 in HAECs. (A)** The genome track of chr16:69412415–69482923 (hg19), a UF-enriched super-enhancer where the H3K27ac signal is increased in HAECs under UF (blue) compared with DF (red). CVD SNP rs75086474 is labeled by an asterisk (*). PCHi-C in HAECs suggested physical interactions between chr16: 69412415–69482923 and the promoters of NAD(P)H:quinone oxidoreductase 1 (NQO1), Cytochrome B5 Type B (CYB5B), and WW Domain Containing E3 Ubiquitin Protein Ligase 2 (WWP2). **(B)** Increased transcriptional levels of NQO1, CYB5B, and WWP2 in HAECs in HAECs under UF compared with those under DF. **(C)** CRISPR interference targeting the SNP rs75086474-surrounded H3K27ac peak reduced the mRNA expression of NQO1, CYB5B, and WWP2 in HAECs cultured under UF. (*n* = 7–10). **(D)** CRISPR activation targeting the SNP rs75086474-surrounded H3K27ac peak increased the mRNA expression of NQO1, CYB5B, and WWP2 in HAECs. (*n* = 4–6). **(E)** CRISPR activation targeting the SNP rs75086474 significantly increased the protein expression of NQO1 and WWP2 in HAECs. (*n* = 3–4). Data represent mean ± SEM. *adjusted P value ≤0.05 and **adjusted P value ≤0.01 were determined by two-sided Student’s *t* test. Source data are available for this figure: SourceData F7.

**Figure S3.**
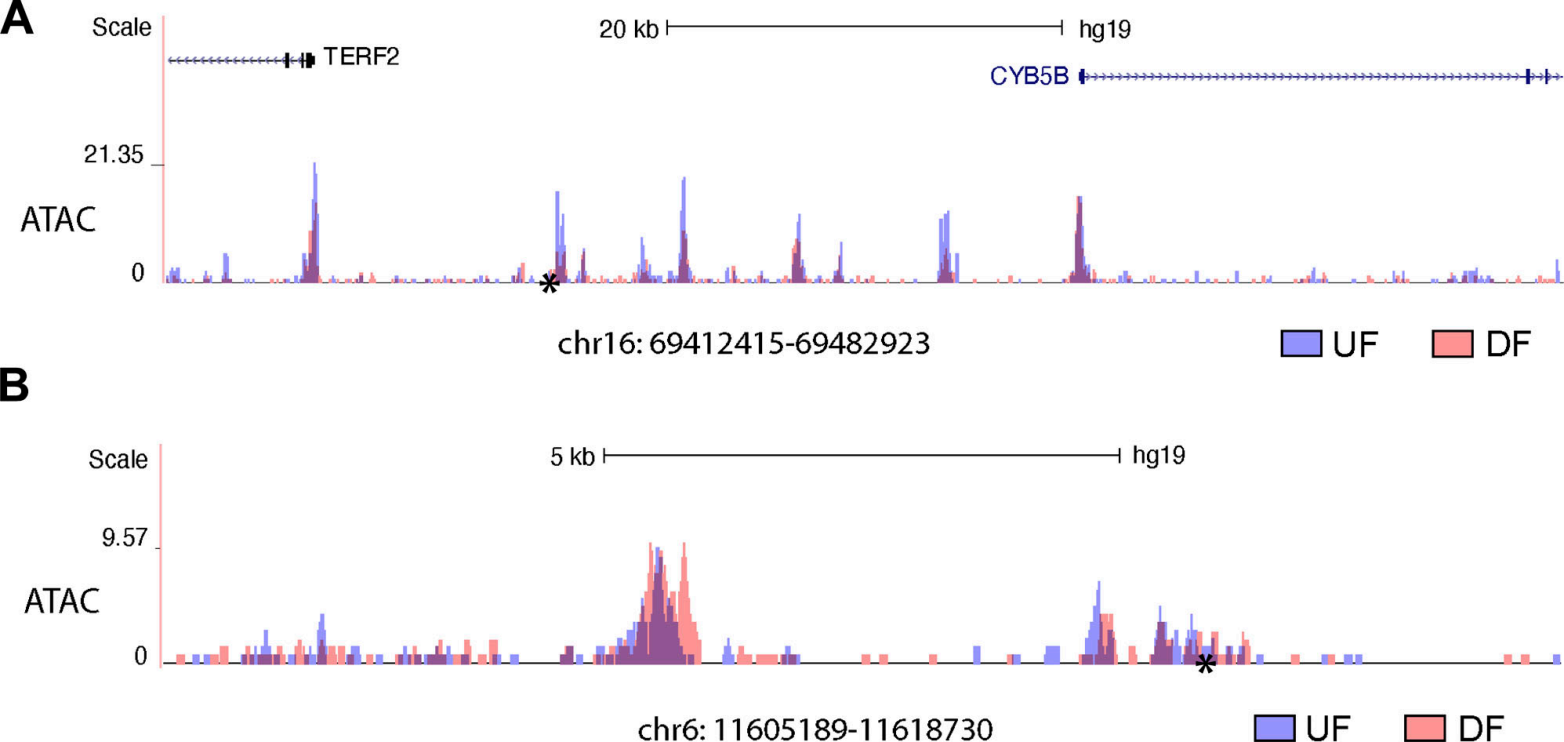
**ATAC-seq demonstrating altered chromatin accessibility of chr16: 69412415–69482923 and chr6: 11605189–11618730 in HAECs cultured under different flow conditions. (A)** Increased chromatin accessibility at chr16: 69412415–69482923 in HAECs subjected to 24-h unidirectional flow (UF) when compared with those under 24-h disturbed flow (DF). CVD SNP rs75086474 is labeled by an asterisk (*). **(B)** Increased chromatin accessibility at chr6: 11605189–11618730 in HAECs subjected to 24-h DF when compared with those cultured under 24-h UF. Thrombosis SNP rs113092656 is labeled by an asterisk (*).

Sequencing-based identification of super-enhancers requires experimental validation (Blobel et al., 2021). Therefore, we set out to determine the regulatory function of UF-enriched SEs in regulating gene expression in HAECs. We prioritized chr16: 69412415–69482923 for investigation based on the following reasons. First, GWAS Catalog has demonstrated that chr16: 69412415–69482923 harbors a genetic variant rs75086474 strongly associated with CVD. Specifically, UK Biobank showed that SNP rs75086474 is significantly associated with CVD (P value = 6E−10; Kichaev et al., 2019) and vascular/heart problems diagnosed by a doctor (P value = 4.7E−10; Neale’s Research Group, 2019). Second, super-enhancer analyses in HAECs under athero-relevant flows showed that the H3K27ac activity at chr16: 69412415–69482923 locus is markedly increased by UF when compared with DF (Fig. 7 A). Third, CHiCAGO-annotated PCHi-C demonstrated that chr16: 69412415–69482923 can physically contact with five promoters in HAECs: polypeptide deformylase (PDF), component of oligomeric Golgi complex 8 (COG8), NAD(P)H:quinone oxidoreductase 1 (NQO1), Cytochrome B5 Type B (CYB5B), and WW Domain Containing E3 Ubiquitin Protein Ligase 2 (WWP2). Fourth, RNA-seq detected three (NQO1, CYB5B, and WWP2) of these five genes were transcriptionally upregulated in HAECs subjected to UF compared with DF (Fig. 7 B). Consistent with the H3K27ac activity, our data of Assay for Transposase-Accessible Chromatin using sequencing (ATAC-seq) demonstrated that the chromatin accessibility at chr16: 69412415–69482923 is increased in HAECs under UF when compared with DF (Fig. S3 A).

Notably, NQO1, CYB5B, and WWP2 not only are all induced by UF but also implicated in the antioxidant endothelial phenotype associated with UF. NQO1 acts both as a quinone reductase and a superoxide reductase, which protects against endothelial inflammation and spontaneous hypertension (Kim et al., 2011; Ross and Siegel, 2021). CYB5B forms a reducing system with cytochrome b5 reductase type 3 (CYB5R3) and NADH to protect against oxidative stress (Siendones et al., 2014). WWP2 modulates the ubiquitination of Septin4 to protect against oxidative stress-induced endothelial injury and vascular remodeling (Zhang et al., 2020).

To test the regulatory role of chr16: 69412415–69482923 in endothelial NQO1, CYB5B, and WWP2 expression, we used CRISPR interference (CRISPRi; Gilbert et al., 2013) to suppress the activity of this cis-regulatory element and its contacted promoters (Fulco et al., 2016). We transduced HAECs with dCas9-KRAB carrying adenovirus, followed by transfection of a pair of sgRNAs targeting the H3K27ac peak surrounding SNP rs75086474. Then we cultured these cells under UF for 24 h before isolating their RNA for RT-qPCR. We found that rs75086474-targeted sgRNAs significantly reduced the mRNA expression of NQO1, CYB5B, and WWP2 compared with cells transfected with non-targeting sgRNA (Fig. 7 C). Moreover, we conducted CRISPR activation (CRISPRa; Chavez et al., 2015) to further activate this cis-regulatory element. We adapted nuclease-null Cas9 conjugated with VP64-p65-Rta (VPR), showing that rs75086474 targeted sgRNAs along with dCas9-VPR significantly increased the mRNA expression of NQO1, CYB5B, and WWP2 in HAECs under static conditions (Fig. 7 D). Moreover, Western blots demonstrated that CRISPRa with rs75086474 targeted sgRNAs significantly increased the protein expression of NQO1 and WWP2 in HAEC (Fig. 7 E). These results validate the regulatory activity of the super-enhancer chr16: 69412415–69482923 in upregulating multiple important antioxidant genes in endothelial cells under UF.

### DF-enriched super-enhancer chr6: 11605189–11618730 upregulates two DF-induced prothrombotic genes EDN1 and HIVEP in HAECs

**Figure 8.**
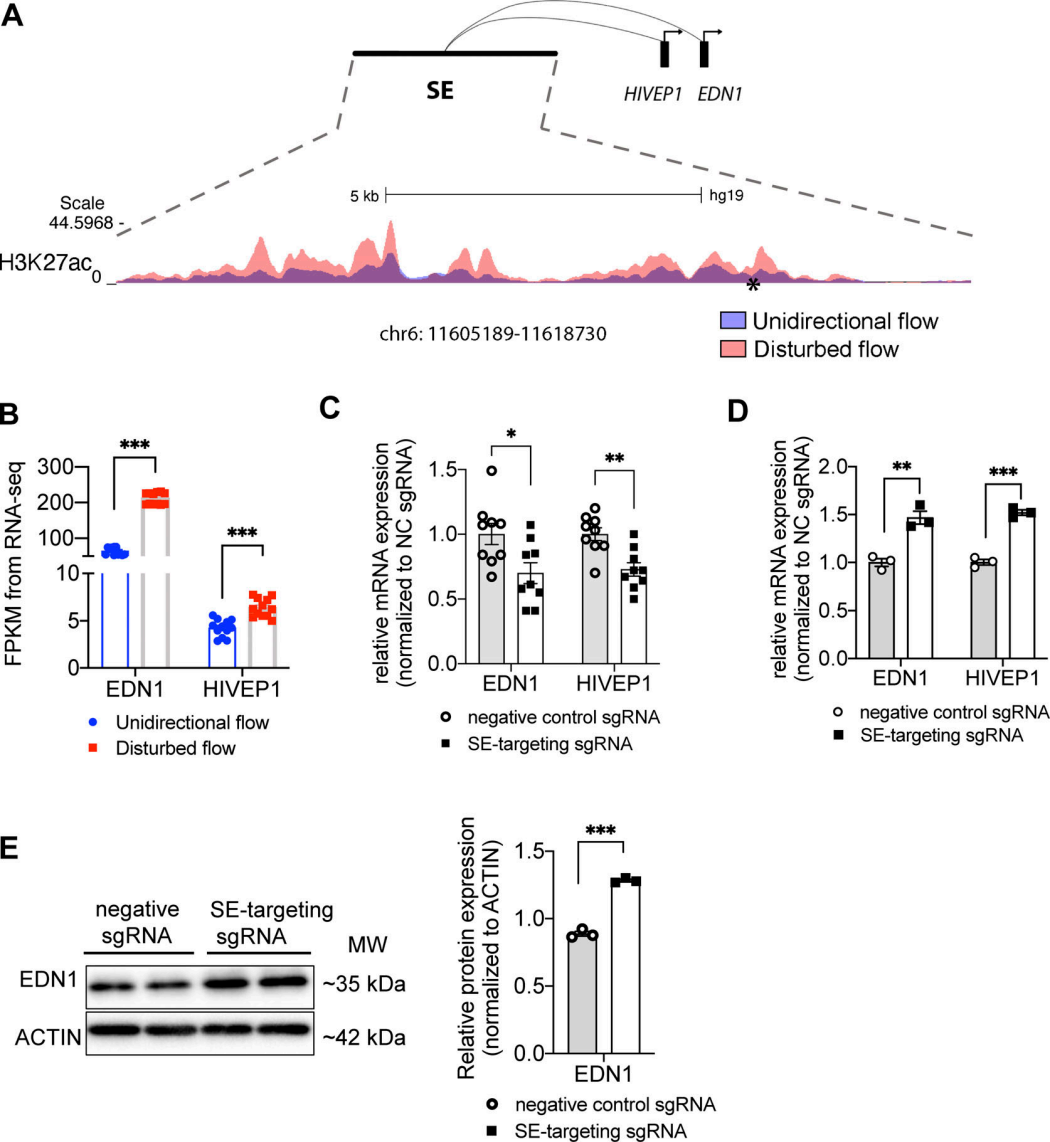
**DF-enriched super-enhancer chr6: 11605189–11618730 upregulates two DF-induced prothrombotic genes EDN1 and HIVEP1 in HAECs. (A)** The genome track of chr6: 11605189–11618730 (hg19), a DF-enriched super-enhancer where H3K27ac signal is increased in HAECs under DF (red) compared with UF (blue). Thrombosis SNP rs113092656 is labeled by an asterisk (*). PCHi-C in HAECs revealed physical interactions between chr6: 11605189–11618730 and the promoters of endothelin-1 (EDN1) and members of the ZAS family, ZAS1 (HIVEP1). **(B)** Increased transcriptional levels of EDN1 and HIVEP1 in HAECs under DF compared with those under UF. **(C)** CRISPR interference targeting the SNP rs113092656-surrounded H3K27ac peak effectively reduced the mRNA expression of EDN1 and HIVEP1 in HAECs cultured under DF. (*n* = 6). **(D)** CRISPR activation targeting the SNP rs113092656-surrounded H3K27ac peak effectively increased the mRNA expression of EDN1 and HIVEP1 in HAECs. (*n* = 3). **(E)** CRISPR activation targeting the SNP rs113092656- significantly increased the protein expression of EDN1 in HAECs. (*n* = 3). Data represent mean ± SEM. *adjusted P value -≤0.05 and **adjusted P value ≤0.01 were determined by two-sided Student’s *t* test. Source data are available for this figure: SourceData F8.

We then prioritized a DF-induced endothelial super-enhancer chr6: 11605189–11618730 for functional investigation. H3K27ac ChIP-seq indicated that its enhancer activity is significantly induced by DF (Fig. 8 A). Chr6: 11605189–11618730 harbors a GWAS SNP rs113092656 that is significantly associated with thrombosis (Hinds et al., 2016). In agreement with the H3K27ac results, our ATAC-seq showed that the chromatin accessibility of chr6: 11605189–11618730 is markedly increased in DF-exposed HAECs when compared with cells under UF (Fig. S3 B). CHiCAGO-analyzed PCHi-C in HAECs demonstrated that chr6: 11605189–11618730 can physically contact with the promoters of endothelin-1 (EDN1) and members of the ZAS family, ZAS1 (HIVEP1). The transcriptional levels of EDN1 and HIVEP1 in HAECs are both significantly induced by DF compared with UF (Fig. 8 B).

Endothelin-1 is primarily produced by endothelial cells and functions as one of the most potent vasoconstrictors in humans (Yanagisawa et al., 1988). EDN1 contributes to CVD and thrombosis through both a paracrine fashion by promoting vascular smooth muscle cell-mediated vasoconstriction and remodeling (Amiri et al., 2004; Yanagisawa et al., 1988), as well as an autocrine mechanism by inhibiting eNOS expression while increasing von Willebrand factor (vWF; Halim et al., 1994; Ramzy et al., 2006). The regulation of EDN1 by a flow-sensitive super-enhancer was not previously proposed. HIVEP1 belongs to the HIVEP family, which are DNA-binding proteins containing several zinc fingers (Baldwin et al., 1990). Increased endothelial HIVEP1 has been linked to elevated endothelial platelet adhesion (Baar, 2019), and genetic variants at the HIVEP1 locus are associated with venous thrombosis (Morange et al., 2010). In addition, plasma HIVEP1 level is positively associated with the occurrence of venous thromboembolism (Bruzelius et al., 2016).

We then conducted a CRISPRi experiment to test the causal role of this DF-induced super-enhancer chr6: 11605189–11618730 in regulating EDN1 and HIVEP1 transcription in HAECs under DF. We designed sgRNAs to target the H3K27ac peak surrounding rs113092656. Our data suggested that compared with the non-targeting sgRNA, rs113092656-targeted sgRNAs along with dCas9-KRAB significantly reduced the transcription of EDN1 and HIVEP1 in HAECs under 24-h DF (Fig. 8 C). dCas9-VPR with rs113092656-targeted sgRNAs significantly increased the mRNA of EDN1 and HIVEP1 in HAECs under static conditions (Fig. 8 D). Moreover, we detected a significant increase of EDN1 protein in HAECs transfected with rs113092656-targeted CRISPRa (Fig. 8 E). H3K27ac ChIP-seq, ATAC-seq, PCHi-C, CRISPRi, and CRISPRa experiments collectively demonstrate that chr6: 11605189–11618730 functions as a DF-induced super-enhancer to promote endothelial expression of EDN1 and HIVEP.

## Discussion

Super-enhancers have emerged as prominent cis-regulatory elements to orchestrate the cell type–specific transcriptome critical to the biological processes in development (Lee et al., 2018) and disease progression (Wilflingseder et al., 2020). Mechanotransduction mechanisms are instrumental to embryonic and organ development as well as the physiological control of tissue homeostasis (Jaalouk and Lammerding, 2009); nevertheless, the molecular identity of mechanosensitive super-enhancers remains poorly understood. Endothelial mechanosensing mechanisms are crucial regulatory controls of vascular homeostasis and diseases (Davies et al., 2013; Fang et al., 2019; Gimbrone and García-Cardeña, 2016; Hahn and Schwartz, 2009; Li et al., 2021). Here, we provide a systematic characterization of typical-enhancers and super-enhancers in endothelial cells subjected to well-defined atherosclerosis-relevant hemodynamic forces by integrating multiomics datasets including H3K27ac ChIP-seq, transcription factor ChIP-seq, PCHi-C, GWAS, and RNA-seq. We report that compared with TEs, SEs are enriched with the binding sites of multiple key endothelial transcription factors and genetic variants associated with cardiovascular diseases. The overall mRNA expression of genes that are physically contacted by SEs is higher compared with genes contacted by TEs. In HAECs, super enhancer–contacted genes are enriched in biological processes related to endothelial specification and vascular functions. We also characterized a cohort of endothelial super-enhancers that are specifically activated by athero-protective unidirectional flow or by athero-prone disturbed flow. Genes contacted by UF-enriched super-enhancers overall have higher transcriptional levels in endothelium under UF whereas those contacted by DF-enriched super-enhancer are largely transcriptionally upregulated by DF. CRISPRi and CRISPRa were employed to demonstrate the regulatory function of one UF-enriched and one DF-enriched SE, both of which harbor a CVD genetic variant and physically contact with the promoters of flow-sensitive genes. To this end, we successfully integrated multilayer omics to systematically characterize the cis-regulatory architecture of the flow-sensitive endothelial epigenome, particularly super-enhancers, as a function of hemodynamic forces instrumental to vascular hemostasis and disease.

Our H3K27ac ChIP-seq results identified 1,000 super-enhancers and 26,457 typical-enhancers in endothelium under blood flows. These results support the notion that super-enhancers represent <5% of the enhancers in a cell (Lovén et al., 2013). Genomic annotation demonstrated that SEs preferentially locate in intragenic regions and distribute less in intergenic and promoter-adjacent regions compared with TEs. The preferred intragenic distribution has also been reported for ATAC-seq-defined SEs in vascular endothelium in culture from human atherosclerotic lesions (Örd et al., 2021). Since the abovementioned study was conducted in isolated blood vessels where the blood flow was absent after the endarterectomy operations, our new results provide a complementary data resource for endothelial enhancer structures shaped by well-defined hemodynamics.

Large-scale human genetics studies have evidently established the association of common genetic variants with human diseases. Recent discoveries demonstrated that disease-associated loci are enriched in tissue-specific regulatory regions, including enhancers in corresponding disease-relevant cell types (Musunuru et al., 2010; Örd et al., 2021). Consistently, GWAS studies on CVD revealed that over 90% of these genetic variants are located in the noncoding genome (Erdmann et al., 2018; Won et al., 2015), and a few candidate CVD variants have been shown to exert biological functions by modulating the activities of cis-regulatory elements, particularly enhancers in cardiovascular-related cells (Stolze et al., 2020). For instance, rs17293632 in the CAD locus 15q24.1 is associated with the chromatin accessibility of an enhancer and consequent SMAD3 expression in human coronary artery smooth muscle cells (Miller et al., 2016). The enhancer variant rs12740374 at CVD locus 1p13 influences low-density lipoprotein cholesterol (LDL-C) levels by regulating sortilin 1 (SORT1) expression in hepatocytes (Musunuru et al., 2010). Here, we showed that endothelial super-enhancers are enriched with CVD SNPs, supporting the emerging importance of genetic contribution to the arterial wall-specific mechanisms in CVD (Howson et al., 2017). Given the critical role of endothelial mechanotransduction in CVD pathogenesis, this study provides a roadmap and public dataset for future studies to identify the causal CVD variants/genes and elucidate the underlying molecular mechanisms, which are ongoing challenges in the post-GWAS era (Erdmann et al., 2018). For instance, CVD SNP rs17114036, located in a flow-sensitive endothelial enhancer, controls the expression of phospholipid phosphatase 3 (PLPP3), key to endothelial quiescence and vessel integrity under UF (Krause et al., 2018; Wu et al., 2015).

Enhancers control the spatial-temporal gene expression through physical contact (Schoenfelder and Fraser, 2019). Analyses of coordinated activation of enhancers and promoters by chromatin accessibility assay without experimentally verified 3D genome organization have been employed to annotate putative active enhancers in vascular cells (Örd et al., 2021). Here, we advanced the endothelial functional genomics studies by conducting the PCHi-C assay. Hi-C was developed to identify the entire ensemble of chromosomal interactions within a cell population and has been conducted in human umbilical vein endothelial cells (HUVECs) and HAECs (Åkerborg et al., 2019; Lalonde et al., 2019; Niskanen et al., 2018). PCHi-C was further developed to specifically map the genome-wide promoter-interacting DNA sequences by enriching the promoter-containing ligation products from Hi-C libraries using tens of thousands of biotinylated RNA 120-mers to pull down fragments containing all annotated promoters (Mifsud et al., 2015; Schoenfelder et al., 2015). Our PCHi-C profiles in HAECs analyzed by CHiCAGO (Cairns et al., 2016) identified the whole-genome 3D ensemble of promoter-interacting regions in human endothelium. The PCHi-C data allow us to further refine the H3K27ac-implicated enhancers by assigning their contacted promoters. Our results demonstrate that SE-contacted genes are enriched in endothelial and vascular biological processes while TF-contacted genes are involved in more general cellular pathways, further supporting the proposed role of super-enhancers in determining the cell type–specific transcriptome (Hnisz et al., 2013; Whyte et al., 2013). Our results show that SE-contacted genes are overall more highly expressed than TE-contacted genes, evidencing the predicted function of SEs to confer stronger activation of their target genes (Hnisz et al., 2013; Lovén et al., 2013). One possible mechanism is that the individual enhancer loci within an SE tend to form concurrent contacts with the given promoter (Fig. 1 J) to exert their regulation to the maximum. Our PCHi-C dataset in HAECs can be used in junction with the Hi-C profiles in HUVECs and HAECs (Åkerborg et al., 2019; Lalonde et al., 2019) to construct a basic architecture of the endothelial 3D genome regardless of stimuli.

Super-enhancers are implicated in functions related to tissue-specific or developmental stage–specific manner (Hnisz et al., 2013; Lee et al., 2018), and our results further demonstrate their dynamic regulation by atherosclerosis-relevant hemodynamic forces. Genes physically contacted by UF-enriched SEs are preferentially upregulated by UF, whereas those contacted by DF-enriched SEs are favorably upregulated by DF, which points to coordinated action of super-enhancer activity and targeted gene transcription as a function of blood flow types. Distinct transcription factor binding motifs were found between the UF-enriched and DF-enriched super-enhancers. These results collectively suggest a plausible mechanism that flow-sensitive endothelial transcriptome, at least partially, is attributable to the combinatorial activation or suppression of enhancers and transcription factors, a model proposed to drive the context-dependent gene regulation required for macrophage specification (Gosselin et al., 2014) and endothelial function (Moonen et al., 2022). The UF-enriched super-enhancers exhibit a notable enrichment of binding sites for ETS-1, a transcription factor known to play a pivotal role in endothelial responses to unidirectional flow (Meadows et al., 2009; Milkiewicz et al., 2008). In contrast, binding sites of proinflammatory transcription factors Jun/AP1 and NFκB-p65 (Fang et al., 2010; Hajra et al., 2000) are enriched in the DF-enriched super-enhancers. This finding is consistent with the previous observation in endothelial cells that a much higher density of p65 motifs was found in TNFα-gained super-enhancers than in TNFα-lost super-enhancers (Brown et al., 2014). These data also support the recently emerging condensate model for gene regulation (Blobel et al., 2021; Sabari et al., 2018; Saravanan et al., 2020) that super-enhancers cooperatively assemble a dynamically regulated dense transcriptional apparatus.

Although epigenetic studies and motif analyses promote the genome-wide discovery of super-enhancers, chromatin and transcription factor profiling alone do not ascertain enhancer activity. Functional assays are critical to verify the enhancer activity and eliminate false discoveries. To do so, we have prioritized two omics-detected flow-sensitive endothelial SEs for functional dissection of their transcriptional regulatory activity on target genes. chr16: 69412415–69482923 is a UF-enriched while chr6: 11605189–11618730 is a DF-induced SE identified by H3K27ac ChlP-seq and ATAC-seq. Both loci contain genetic variants associated with CVD whereas their enhancer activities have not been experimentally determined. The activities of these two flow-sensitive SEs are supported by PCHi-C, indicating their physical looping to promoters of flow-regulated genes. In agreement with the chromatin profiling and 3D endothelial genome architecture, CRISPR interference and activation experiments demonstrated that the UF-enriched SE chr16: 69412415–69482923 is critical to the elevated expression of NQO1, CYB5B, and WWP2 in endothelial cells under UF. Meanwhile, DF-enriched SE chr6, 11605189–11618730, regulates the increased endothelial expression of EDN1 and HIVEP1 under DF. Experimental validation of these two genomic loci further supports the notion that a single super-enhancer can impact a complex regulatory network and consequent physiological processes by coordinately activating functionally connected genes, a model proposed in other gene regulatory mechanisms such as microRNAs (Fang et al., 2010; Fang and Davies, 2012). The functions of NQO1, CYB5B, and WWP2 in promoting the antioxidant endothelial phenotype have been well established (Chen et al., 2003; Siendones et al., 2014; Zhang et al., 2020), whereas EDN1 and HIVEP1 are implicated in the proinflammatory and prothrombotic endothelial function (Amiri et al., 2004; Baar, 2019; Bruzelius et al., 2016; Ramzy et al., 2006; Yanagisawa et al., 1988). The presence of the respective CVD-associated SNPs (rs75086474 and rs113092656) in these two flow-sensitive SEs suggests a possible convergence of CVD genetic predisposition and mechanotransduction mechanisms on enhancer activities, a phenomenon that has been demonstrated for CVD SNP rs17114036 (Krause et al., 2018). Nevertheless, whether rs75086474 and rs113092656 are the causal SNPs and the plausible underlying molecular mechanisms remain unknown. This will be the subject of future studies. Furthermore, the mechanoregulation of endothelial enhancers by additional mechanical cues, such as cyclic stretch, in diverse vascular beds, including microvasculature (Huang et al., 2017; Wu et al., 2021), remains to be investigated.

Cellular transcriptional responses to biomechanical stimuli are critical to embryogenesis, organ development, and pathophysiological control of tissues. The endothelial transcriptome is tightly and dynamically regulated by blood flow, which is a major regulator of vascular network morphogenesis, vascular tone control, vessel structure, and localization of pathological vascular remodeling. Atherosclerosis and stenosis largely initiate and develop in arterial regions where the local disturbed flow activates endothelial cells, whereas unidirectional flow promotes the anti-inflammatory and antioxidant endothelial phenotype. By employing multilayer omics approaches, we systematically characterized the chromatin architecture, particularly enhancers, in endothelial cells under well-defined hemodynamics. In summary, our results elucidate the identity and highlight the importance of super-enhancers as CVD SNP-enriched cis-regulatory elements contributing to the flow-regulated endothelial transcriptome key to vascular health and disease.

## Materials and methods

### Application of athero-relevant flows in vitro

A cone and plate flow device consisting of a computerized stepper motor UMD-17 (Arcus Technology), and a 1° tapered stainless steel cone was used to generate the physiologically relevant shear stress patterns. The flow device was placed in a 37°C incubator with 5% CO_2_. Human aortic endothelial cells (HAECs, CC-2535; Lonza) at 100% confluence, maintained in EGM-2 medium containing 4% dextran in six-well plates, were subjected to unidirectional flow (UF) or disturbed flow (DF) for 24-h before harvesting. The disturbed flow here recreates the hemodynamics measured in human carotid sinus prone to atherogenesis and unidirectional flow and represents the hemodynamics measured in human distal internal carotid artery resistant to atherosclerosis (Dai et al., 2004). Static cells used the same media above and did not utilize the flow devices.

### H3K27ac chromatin immunoprecipitation with whole genome sequencing (ChIP-seq) and RNA-seq

H3K27ac ChIP-seq and RNA-seq were conducted in low-passage human aortic endothelial cells (HAECs; Lonza) subjected to 24-h “athero-prone” disturbed flow mimicking the hemodynamics measured in the human carotid sinus or “athero-protective” unidirectional representing the wall shear stress in human distal internal carotid artery.

For ChIP-seq, cells were washed three times with warm PBS and then trypsinized. Cells were pelleted at 3,000 × *g* for 5 min before being fixed at room temperature with 1% paraformaldehyde in PBS for 10 min and quenched with 125 mM glycine. One million cells were used for each ChIP-seq. Cell lysates were sonicated using BioRuptor Pico (Diagenode) and then immunoprecipitated using antibodies against H3K27ac (39135; Active Motif) or H3K4me2 (07–030; EMD Millipore), bound to a 2:1 mixture of Protein A Dynabeads (10002D; Invitrogen) and Protein G Dynabeads (10004D; Invitrogen). Following immunoprecipitation, crosslinking was reversed and libraries were prepared beginning with dsDNA end repair and excluding UDG. For each sample condition, an input library was also created using an aliquot of sonicated cell lysate that had not undergone immunoprecipitation. These samples were sequenced on an Illumina HiSeq 4000 and used to normalize ChIP-seq results.

For RNA-seq, cells were washed three times with warm PBS, and total RNA was then isolated in Trizol using Direct-zol RNA MiniPrep kit (R2053; Zymo) with in-column DNaseI digestion. The RNA quality was assessed by 2100 Bioanalyzer (Agilent). Sequencing was done on Illumina HiSeq 2500 with paired-end 75 bp read length.

### ChIP-seq analysis and identification of super-enhancers and typical-enhancers

Using Cutadapt (Martin, 2011), adapters were trimmed from the raw sequencing data of H3K27ac ChIP-seq. Reads were then aligned to the UCSC hg19 genome using Bowtie2 (Langmead and Salzberg, 2012; version 2.3.4.3) with default parameters to generate SAM files. SAM files were filtered and converted to BAM files, followed by sorting and PCR duplicate removal using SAMtools (Li et al., 2009; version 1.6.0). Mapped reads were then organized into tag directories using the “makeTagDirectory” command of HOMER (Heinz et al., 2010; version 4.10.0). Tag directories of five biological replicates from unidirectional flow were merged for IP and input samples, respectively. Similarly, tag directories of six biological replicates from disturbed flow were merged for IP and input samples, respectively.

Super-enhancers and typical-enhancers were identified using HOMER “findPeaks -style super -L 0,” in the merged IP tag directories against the input tag for each experimental flow condition. In brief, super-enhancers and typical-enhancers were classified following the criteria established by the Young laboratory (Whyte et al., 2013). H3K27ac-implicated enhancers within 12.5 kb of each other were stitched together to define a single entity and ranked along the x-axis in the ascending order of H3K27ac signals plotted on the y-axis. Super-enhancers are defined as those to the right of the tangent point (slope = 1) of the resulting curve. The remaining enhancer regions are designated as typical-enhancers. HOMER “mergePeaks -d given” was used to combine the super-enhancers in HAECs exposed to two types of flows, which added up to 1,000 endothelial super-enhancers, and similarly for the identification of 26,457 typical-enhancers. To plot the heatmaps of H3K27ac distribution, we generated the data matrix using HOMER “annotatePeaks.pl -ghist.” Each enhancer together with the 10,000 bp surrounding its center was divided into 200 U, and the number of tags mapped to each unit was counted. Within each super- or typical-enhancer, the unit with the maximum number of tag counts was aligned to the middle point of the x-axis for visualization using MATLAB “heatmap” function. Histograms were generated by taking the average of tag counts across all units at each enhancer locus.

To identify unidirectional flow (UF)- and disturbed flow (DF)-enriched endothelial super-enhancers, HOMER “getDifferentialPeaks -F 1.2” was used to detect super-enhancers that have a ≥1.2-fold change of normalized tag count in HAECs in comparison between the UF and DF conditions (Poisson enrichment P value ≤1e−4). The complementary set of super-enhancers out of the 1,000 were defined as core super-enhancers.

### Motif enrichment analysis

Motif enrichment analysis was performed using HOMER “findMotifGenome.pl” command in UF-enriched, DF-enriched, and core super-enhancers, respectively. Option “-size given” was specified to find motifs using the exact size of each super-enhancer. HOMER randomly selected both size-matched and GC content-matched genomic regions as background and referred to them to discover enriched motifs in each type of super-enhancers.

### Counting enhancers with transcription factor binding sites

ChIP-seq of transcription factors EGR, JUN, JUNB, or NFκB-p65 conducted in HAECs (Hogan et al., 2017) were employed to map the transcription factor binding sites in endothelial super-enhancers and typical-enhancers identified in this study. To evaluate the proportions of super-enhancers and typical-enhancers that contain a transcription factor binding site, the normalized tag counts of each TF ChIP-seq along every SE and TE were calculated using HOMER “annotatePeaks.pl.” For each TF ChIP-seq, the numbers of SEs and TEs that contain non-zero normalized tag counts were added up, which were then divided by the total numbers of SEs (1,000) and TEs (26,457), respectively, to calculate the percentage. To examine the TF binding site density, the previously calculated normalized tag counts along each SE and TE were further divided by the enhancer length in bp.

### PCHi-C and enhancer annotation

PCHi-C was conducted in HAECs for a three-dimensional (3D) genome-wide detection of endothelial promoter-interacting cis-regulatory elements. Specifically, in situ Hi-C was performed as described by Rao et al. (2014). Five million HAECs were harvested from the culture and then resuspended in 1× DPBS. To crosslink interacting DNA loci, 37% formaldehyde was added to the cells to a final concentration of 1% and carried out for 10 min at room temperature. Crosslinked chromatin was digested using MboI endonuclease (R0147; New England Biolabs) to generate ligation fragments, ∼400–500 bp, allowing enhancer-level resolution of promoter contacts; the restriction overhangs were filled in and the DNA ends were marked with biotin-14-dATP (19524-016; Life Technologies). To isolate captured fragments, the biotin-labeled DNA was then sheared and pulled down using Dynabeads MyOne Stretavidin T1 beads (65602; Life Technologies). The in situ Hi-C library was amplified off the T1 beads with six cycles of PCR using Illumina primers (Illumina, 2007). The promoter-containing fragments were further isolated from the whole-genome Hi-C library as described by Montefiori et al. (2018). Specifically, the Hi-C library was hybridized to 81,735 biotinylated 120-mer custom RNA oligomers (CustomArray, Inc.) targeting 22,600 human RefSeq protein-coding promoters (four probes/RefSeq transcription start sites) and added to streptavidin-coated magnetic beads (65602; Life Technologies). Subsequently, an eight-cycle PCR was performed to amplify the DNA bound to the beads captured by the biotinylated RNA. Each library was sequenced on a full lane of an Illumina HiSeq 4000 machine.

Capture Hi-C analysis was then performed using HOMER. Specifically, Hi-C reads were aligned and filtered using HiCUP (Wingett et al., 2015). The computational pipeline CHiCAGO (Cairns et al., 2016), which eliminates sequence capture bias, was employed to identify genomic interactions. Only strong interactions with CHiCAGO scores ≥5 were selected for further analysis. Promoter-interacting regions from PCHi-C were then overlapped with H3K27ac-identified endothelial typical-enhancers and super-enhancers, and intersections were selected using HOMER “mergePeaks -prefix” and assigned back to their interacted promoters for further gene annotation.

The number of interactions that an SE or TE can form with promoters was calculated by counting the number of overlaps of an SE or TE with the promoter-interacting regions detected in PCHi-C. The number of repetitive interactions of SEs or TEs with the same promoter was calculated as enhancer-gene contact frequency.

### GWAS SNP enrichment analysis

The enrichment of disease-associated variants in endothelial super-enhancers and typical-enhancers was examined using R package traseR (Chen and Qin, 2016). SNP-trait associations were obtained from the combination of dbGaP and NHGRI GWAS Catalogs, and SNPs in linkage disequilibrium (LD, r^2^ > 0.8) within 100 kb of the lead SNPs were obtained from 1,000 Genome Project, which generated 78,247 unique LD trait-associated SNPs. Whole genome outside the enhancer regions was referred to as the background. The significant enrichment was examined using the binomial test, with the null hypothesis being the probability of observing a base to be trait-associated SNP is the same in the enhancer regions as in their corresponding background regions. The test was performed for 33 GWAS classes and 573 GWAS traits. A statistical q value <0.05 and odds ratio >1 were used as the threshold cutoffs for significant enrichment.

### Refining super-enhancers that contain cardiovascular diseases (CVD)-associated GWAS SNP

The CVD GWAS traits were curated using the following keywords: cardiovascular (for cardiovascular disease), coronary artery/coronary heart (for coronary artery and heart disease), myocardial infarction (for myocardial infarction), atherosclerosis/plaque (for atherosclerosis), thrombo (for thrombosis), stroke (for stroke), heart failure/heart disease/heart defect (for heart disease), ventricular (for ventricular disease), atrial fibrillation (for atrial fibrillation), blood pressure (for blood pressure), hypertension (for hypertension), valve (for valve defect), carotid (for carotid artery disease), and peripheral (for peripheral artery disease). CVD-associated SNPs were downloaded from NHGRI-EBI GWAS Catalog (Buniello et al., 2019; version 1.0.2), and the genomic coordinates were converted to hg19 using liftOver (Kuhn et al., 2013). The BEDTools intersect (Quinlan and Hall, 2010) was used to refine CVD SNP-containing super-enhancers.

### Gene expression annotation using RNA-seq

RNA-seq results in HAECs subjected to well-defined hemodynamic forces were conducted and analyzed as described previously (Krause et al., 2018). The quality of reads was assessed using fastQC. Reads were aligned to GENCODE hg38.p2 reference genome using Tophat2 version 2.1.1. Transcripts were assembled using the bam files from Tophat2 using Cufflinks version 2.1.1. The transcript files from cufflinks were merged using Cuffmerge. Cuffquant was used to estimate abundances, prior to analysis by Cuffdiff, to estimate differential gene expression. The fragments per kilobase of transcript per million mapped reads (FPKM) were used as the proxy for gene expression level in HAECs subjected to unidirectional flow or disturbed flow. The expression level of genes contacted to super-enhancers was compared with that of genes exclusively contacted to typical-enhancers; the FPKM under unidirectional flow and disturbed flow was averaged for each gene. For mechanosensitive super enhancers–contacted genes, the expression fold change of each gene in response to distinct hemodynamics was calculated as the ratio of its FPKM under unidirectional flow to its FPKM under disturbed flow and represented in a log_2_ scale.

### Gene ontology analyses

Metascape (Zhou et al., 2019) was used to identify enriched biological pathways of selected gene sets. Genes with promoters contacted to super-enhancers or exclusively contacted to typical-enhancers were further filtered to select only genes that were actively expressed (FPKM ≥ 12 in RNA-seq) in HAECs. A similar procedure was followed to identify enriched biological pathways of the genes exclusively contacted to UF-enriched SEs and genes exclusively contacted to DF-enriched SEs, with less stringent criteria to select actively expressed genes (FPKM ≥ 1 in RNA-seq).

### CRISPR interference, CRISPR activation, and RT-qPCR

Catalytically dead Cas9 (dCas9) was fused either to KRAB repressor (cat #46911; Addgene) or to VP64-p65-Rta (VPR; cat #63798; Addgene) and transduced to HAECs using adenoviruses custom-made by Vector Biolabs. Briefly, dCas9-BFP-KRAB or dCas9-VPR-tagBFP was subcloned into pDual2-V5HisbGH vector. The backbone of the viruses is Human Type 5 (dE1/E3) adenoviruses. 1 d after the dCas9-KRAB or dCas9-VPR transduction, HAECs were transfected with two to three targeted sgRNAs (IDT, sequence shown below) or negative control sgRNAs (1072544; IDT) using RNAiMAX (Life Technologies) and cultured in EGM-2 medium (Lonza) containing 4% dextran (for the following flow experiments). Non-targeting negative control guide RNA was purchased from IDT. SE-targeted sgRNAs were specifically designed on IDT to align to the CVD SNP-containing H3K27ac peaks. HAECs were then subjected to unidirectional flow or disturbed flow for 24 h before RNA isolation.

sgRNA #1 targeting UF-enriched super-enhancer of chr16_69412415–69482923: 5′-GTG​TCT​ACA​CCC​CAG​AAA​TG-3′.

sgRNA #2 targeting UF-enriched super-enhancer of chr16_69412415–69482923: 5′-AAT​AAA​CTG​GTG​GGG​AAC​CG-3′.

sgRNA #1 targeting DF-enriched super-enhancer chr6_11605189–11618730: 5′-ACT​AGT​TTC​TTA​GGC​CCA​AC-3′.

sgRNA #2 targeting DF-enriched super-enhancer chr6_11605189–11618730: 5′-ACC​GAG​GGA​AGT​GCT​ACC​AC-3′.

sgRNA #3 targeting DF-enriched super-enhancer chr6_11605189–11618730: 5′-CTG​CCA​GTA​ATT​TAC​GGA​GC-3′.

RNA was isolated from cells using NucleoZOL RNA isolation kits (Takara) and reverse transcribed using High-Capacity cDNA Reverse Transcription Kit (Thermo Fisher Scientific). Quantitative mRNA expression was determined by RT-qPCR using SYBR Green MasterMix (Roche). The following primer (IDT) sequences were used.

β-Actin: 5′-TCC​CTG​GAG​AAG​AGC​TAC​GA-3′ 5′-AGG​AAG​GAA​GGC​TGG​AAG​AG-3′.

GAPDH: 5′-TGC​ACC​ACC​AAC​TGC​TTA​GC-3′ 5′-GGC​ATG​GAC​TGT​GGT​CAT​GAG-3′.

Ubiquitin: 5′-ATT​TAG​GGG​CGG​TTG​GCT​TT-3′ 5′-TGC​ATT​TTG​ACC​TGT​TAG​CGG-3′.

NQO1: 5′- GGC​AGA​AGA​GCA​CTG​ATC​GTA-3′ 5′- TGA​TGG​GAT​TGA​AGT​TCA​TGG​C-3′.

CYB5B: 5′-ATG​TCC​GGT​TCA​ATG​GCG​AC-3′ 5′-CAT​GGA​TCA​CAA​GCC​ACA​GTT-3′.

WWP2: 5′-CAA​AGC​CCA​AGG​TGC​ATA​ATC​G-3′ 5′-CCA​ATG​CGC​TTC​CCA​GTC​T-3′.

EDN1: 5′-AGA​GTG​TGT​CTA​CTT​CTG​CCA-3′ 5′-CTT​CCA​AGT​CCA​TAC​GGA​ACA​A-3′.

HIVEP1: 5′-GAA​CTT​CGG​AAT​CCC​TTA​AAG​GT-3′ 5′-AAG​AAC​GGC​GAA​AGA​TGA​CTC-3′.

### Protein isolation and Western blots

Cells were lysed in 1 × SDS-PAGE sample buffer (0.2 M Tris–HCl, pH 6.8, 8% SDS, 0.1% bromophenol blue, 40% glycerol, 20% β-mercaptoethanol; all chemicals are from Sigma-Aldrich). The protein lysates were centrifuged at 4°C for 10 min at 10,000 × *g* and then boiled at 95°C for 10 min. Protein extracts were separated on 10% SDS-PAGE gel and transferred to a PVDF membrane (BioRad Laboratories). Blots were blocked in TBST with 5% BSA for 1 h, followed by overnight incubation with a primary antibody at 4°C. The primary antibodies used were NQO1 (#11451-1-AP; Proteintech), WWP2 (#15469-1-AP; Proteintech), EDN1 (#ab2786; Abcam), and β-actin (#3700S; Cell signaling). Blots were further incubated with HRP-conjugated anti-mouse secondary antibody (#401253; Sigma-Aldrich) at room temperature for 1 h. Protein bands were developed with the ECL system (Pierce) and performed by ChemiDoc MP Imager (Bio-rad Laboratories). The protein band density was analyzed using ImageJ.

### Online supplemental material

Fig. S1 demonstrates that endothelial super-enhancers are enriched with EC transcription factor binding sites and cardiovascular disease (CVD) GWAS SNPs, and they physically contact with more promoters compared with typical-enhancers. Fig. S2 is a Circos plot illustrating the genome-wide loci contacted by flow-sensitive super-enhancers and the flow-sensitive transcriptome in HAECs. Fig. S3 is the ATAC-seq track of super-enhancers chr16: 69412415–69482923 and chr6: 11605189–11618730. Table S1 lists the 1,000 super-enhancers, 26,457 typical-enhancers, and the strong interactions (CHiCAGO score ≥5) detected by PCHi-C. Table S2 lists the mechano-sensitive super-enhancers and the 34 refined super-enhancers.

## Supplementary Material

Table S1 lists the 1,000 super-enhancers, 26,457 typical-enhancers, and the strong interactions (CHiCAGO score ≥5) detected by PCHi-C.

Table S2 lists the mechanosensitive super-enhancers and the 34 refined super-enhancers.

SourceData F7 is the source file for Fig. 7.

SourceData F8 is the source file for Fig. 8.

## Data Availability

The H3K27ac ChIP-seq and ATAC-seq data have been deposited in the Gene Expression Omnibus (GEO) database: https://www.ncbi.nlm.nih.gov/geo/query/acc.cgi?acc=GSE112340. The RNA-seq data have been deposited in Zenodo: https://doi.org/10.5281/zenodo.260122.
