# Supplementary figures and images for: Mechanosensitive super-enhancers regulate genes linked to atherosclerosis in endothelial cells
Source: J Cell Biol. 2024 Jan 17;223(3):e202211125. doi: 10.1083/jcb.202211125 (PMC10794123; doi:10.1083/jcb.202211125)

SourceDataF7

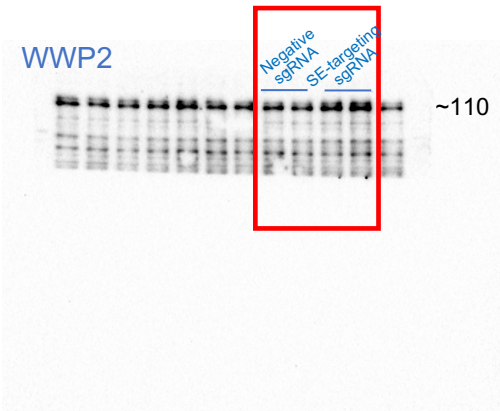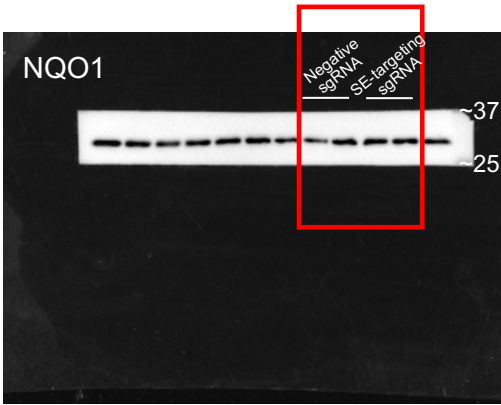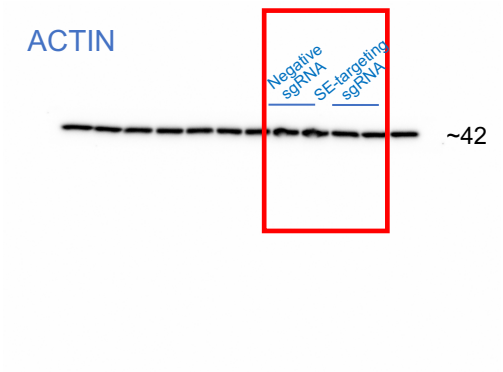

Supplement: SourceData F7 — is the source file for Fig. 7. [file JCB_202211125_SourceDataF7.pdf]

SourceDataF8

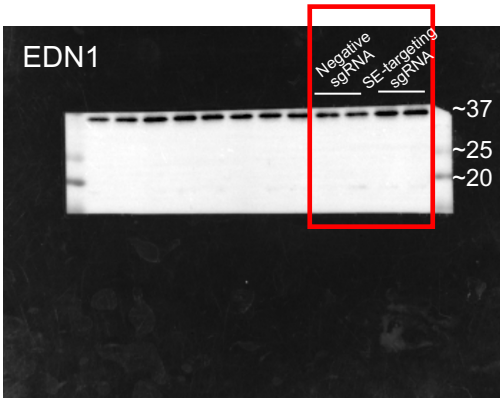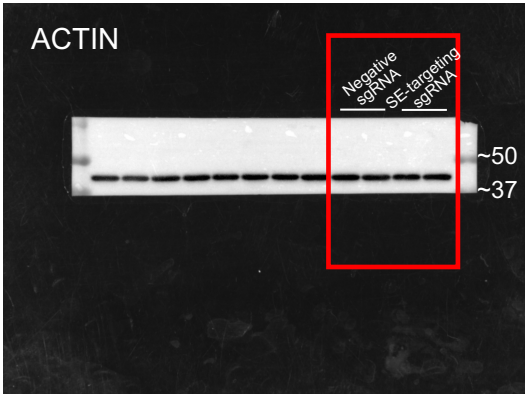

Supplement: SourceData F8 — is the source file for Fig. 8. [file JCB_202211125_SourceDataF8.pdf]
